# CDK7-YAP-LDHD axis promotes D-lactate elimination and ferroptosis defense to support cancer stem cell-like properties

**DOI:** 10.1038/s41392-023-01555-9

**Published:** 2023-08-16

**Authors:** Mengzhu Lv, Ying Gong, Xuesong Liu, Yan Wang, Qingnan Wu, Jie Chen, Qingjie Min, Dongyu Zhao, Xianfeng Li, Dongshao Chen, Di Yang, Danna Yeerken, Rui Liu, Jinting Li, Weimin Zhang, Qimin Zhan

**Affiliations:** 1 Key Laboratory of Carcinogenesis and Translational Research (Ministry of Education), Department of Molecular Oncology, Peking University Cancer Hospital and Institute, Beijing, 100142 China; 2 Key Laboratory of Carcinogenesis and Translational Research (Ministry of Education), Department of Breast Oncology, Peking University Cancer Hospital and Institute, Beijing, 100142 China; 3 Research Unit of Molecular Cancer Research, Chinese Academy of Medical Sciences, Beijing, 100021 China; 4 Peking University International Cancer Institute, Beijing, 100191 China; 5 Institute of Cancer Research, Shenzhen Bay Laboratory, Shenzhen, 518107 China; 6 Department of Oncology, Cancer Institute, Peking University Shenzhen Hospital, Shenzhen Peking University-Hong Kong University of Science and Technology (PKU-HKUST) Medical Center, Shenzhen, 518036 China; 7 Soochow University Cancer Institute, Suzhou, 215127 China

**Keywords:** Cancer metabolism, Cancer stem cells

## Abstract

Reprogrammed cellular metabolism is essential for maintaining cancer stem cells (CSCs) state. Here, we report that mitochondrial D-lactate catabolism is a necessary initiating oncogenic event during tumorigenesis of esophageal squamous cell carcinoma (ESCC). We discover that cyclin-dependent kinase 7 (CDK7) phosphorylates nuclear Yes-associated protein 1 (YAP) at S127 and S397 sites and enhances its transcription function, which promotes D-lactate dehydrogenase (LDHD) protein expression. Moreover, LDHD is enriched significantly in ESCC-CSCs rather than differentiated tumor cells and high LDHD status is connected with poor prognosis in ESCC patients. Mechanistically, the CDK7-YAP-LDHD axis helps ESCC-CSCs escape from ferroptosis induced by D-lactate and generates pyruvate to satisfy energetic demands for their elevated self-renewal potential. Hence, we conclude that esophageal CSCs adopt a D-lactate elimination and pyruvate accumulation mode dependent on CDK7-YAP-LDHD axis, which drives stemness-associated hallmarks of ESCC-CSCs. Reasonably, targeting metabolic checkpoints may serve as an effective strategy for ESCC therapy.

## Introduction

Cancer stem cells are small dormant cancer cell subtype that takes responsibility for tumor initiation, growth, metastasis, recurrence, and chemotherapy resistance.^1^ Previous literatures suggest that CSCs originate from their progenitor cells and depend on specific stem-cell pathways activation^2^ and inactivation of stem-cell self-renewal inhibitory pathways.^3^ Under different selection pressures, genetically distinct CSCs can evolve into diverse subclones in parallel, resulting in tumor heterogeneity.^4^ Although there remain several unknown factors about the regulatory mechanism of CSCs traits, clinical studies have been conducted to eliminate CSCs by targeting Wnt, NOTCH, Hedgehog, Hippo, and other pathways for tumor-initiating activities.^5^ Based on the growing understanding of CSCs’ biological characteristics, these pioneer findings propose that tailored anti-CSCs targeting therapeutic strategies can provide more efficient approaches for clinical practices while reducing drug resistance and tumor relapse.

The cell cycle is fundamentally associated with CSCs self-renewal and differentiation, fine-tuned by different cyclins and cyclin-dependent kinases (CDKs). Recent literature indicates that CDK1 communicates with SOX2 and supports CSCs’ properties in human melanoma^6^ and that CDK4/6 maintains stem cell-like phenotypes in breast cancer,^7^ lung cancer, and ovarian cancer.^8^ Interestingly, growing data suggests that CDKs not only modulate cell cycle but are also linked with cell cycle-independent mechanisms, including repair of damaged DNA, metabolism, epigenetics, and transcription,^9^ demonstrating that CDKs have more extensive roles. Evolutionary studies suggest that CDK1, CDK2, CDK4, and CDK6 are critical cell cycle-related CDKs, while CDKs such as CDK7, CDK8, CDK9, and CDK11 play crucial roles in both cell cycle and transcriptional processes as transcriptional regulatory cofactors.^10,11^ Blocking CDK7 has been implicated in CSCs in thyroid tumors, urothelial carcinoma and pancreatic cancer by attenuating NOTCH1-cMYC, Hedgehog, or the CDK7-dependent transcriptional addictions.^12–14^ In addition, the CDK8-c-MYC axis has been demonstrated to govern the self-renewal potential of CSCs in glioma. There is additional evidence that CDK9 and CDK12 regulate the CSCs’ stemness-associated properties via transcriptional functions.^15,16^ Both cell cycle-related CDKs and transcriptional-related CDKs are involved in CSCs regulation, thus understanding the role of CDKs in CSCs biology is challenging. The current molecular mechanism by which CDKs modulate CSCs properties is still largely incomplete, if cell cycle-dependent or independent function of CDKs is essential for CSCs to maintain their stemness needs further investigation. Therefore, systematic comparison and evaluation of CSCs’ reliance on these CDKs will aid in providing deeper molecular insights and formulating effective strategies to target CDKs to eliminate CSCs.

CSCs’ metabolic features are distinct from those of bulk tumor cells.^17,18^ CSCs rely more on mitochondrial oxidative phosphorylation (OXPHOS) and fatty acid oxidation, whereas differentiated tumor cells depend mainly on glycolysis.^19,20^ However, several studies revealed that CSCs could also use glycolysis and glutaminolysis to sustain energetic demands,^21,22^ and could switch from OXPHOS to glycolysis under metabolic stress,^23^ indicating the metabolic flexibility of CSCs. As mentioned above, CDKs are not only vital in maintaining the stemness of CSCs but they are also involved in the regulation of cellular metabolic processes. CDK6/Cyclin D3 has been shown to phosphorylate and inactivate PFK1 and PKM2, thereby repressing glycolysis in the G_1_ phase,^24^ while CDK6/Cyclin D1 has been shown to suppress the transcriptional activity of PGC-1α and NRF1 to attenuate OXPHOS by downregulating the expression of mitochondrial genes in the G_1_ phase.^25^ Moreover, in the S phase, CDK2/Cyclin E may phosphorylate IDH1/2 and block the tricarboxylic acid (TCA) cycle, whereas CDK2/Cyclin A could repress IDH1/2 in the G_2_ phase.^25^ Besides, CDK1/Cyclin B1 promoted the G_2_/M transition by enhancing OXPHOS.^26^ However, most of the previous studies on CSCs were focused on the abnormal expression or altered activity of metabolic enzymes, and oncogenic-driven metabolic pathway rewiring, leaving little supporting evidence for the role of CDKs-coupled metabolic regulation in CSCs function.

The goal of this work was to identify the key CDKs that regulate CSCs and reveal the underlying metabolic dependency under this scenario. We found that small molecule inhibitor targeting CDK7 elicited powerful CSCs eradication effect at low dose in cell-line CSCs models of ESCC, which suggests that CDK7 is crucial in the regulation of stem cell-like hallmarks in ESCC. Subsequently, we discovered that CDK7 could promote ESCC-CSCs phenotypes by upregulating YAP expression and then facilitating its nuclear phosphorylation at S127 and S397 sites, which could govern transcription driven by YAP, and activated YAP enhanced LDHD expression to accelerate D-lactate catabolism in the mitochondria of ESCC CSCs, ultimately protecting these cells from ferroptosis. These events simultaneously increased the levels of pyruvate generated by D-lactate, which in turn enhanced the stem cell-like properties of esophageal CSCs. These findings demonstrate for the first time that the CDK7-YAP-LDHD axis could shift between the metabolic phenotypes and stemness-associated functions in differentiated ESCC cells and ESCC CSCs and raise the possibility that targeting the CDK7-YAP-LDHD axis metabolic checkpoint might be efficient for treating ESCC patients.

## Results

### CDK7 inhibitor THZ1 shows potent anti-CSCs properties identified by a small-molecule screening

To elucidate the mechanism that modulates CSCs stemness, a sphere formation analysis was first carried out on esophageal squamous cancer cell lineage to determine their self-renewal capability. KYSE410 cells indicated substantially enhanced sphere-forming ability with subsequent serial propagations, suggesting their elevated self-renewal ability (Supplementary Fig. 1a–c). CD90 has been indicated to functionally determine the ESCC-CSCs subset.^27^ Consistently, we found that the spheres formed by KYSE410 cells had a higher CD90 enrichment than those adherent cells (Supplementary Fig. 1d, e). Moreover, the spheres showed markedly enhanced expression of ABCG2, SOX9, SOX2, OCT4, and NANOG relative to adherent monolayers (Supplementary Fig. 1f, g).

**Fig. 1:**
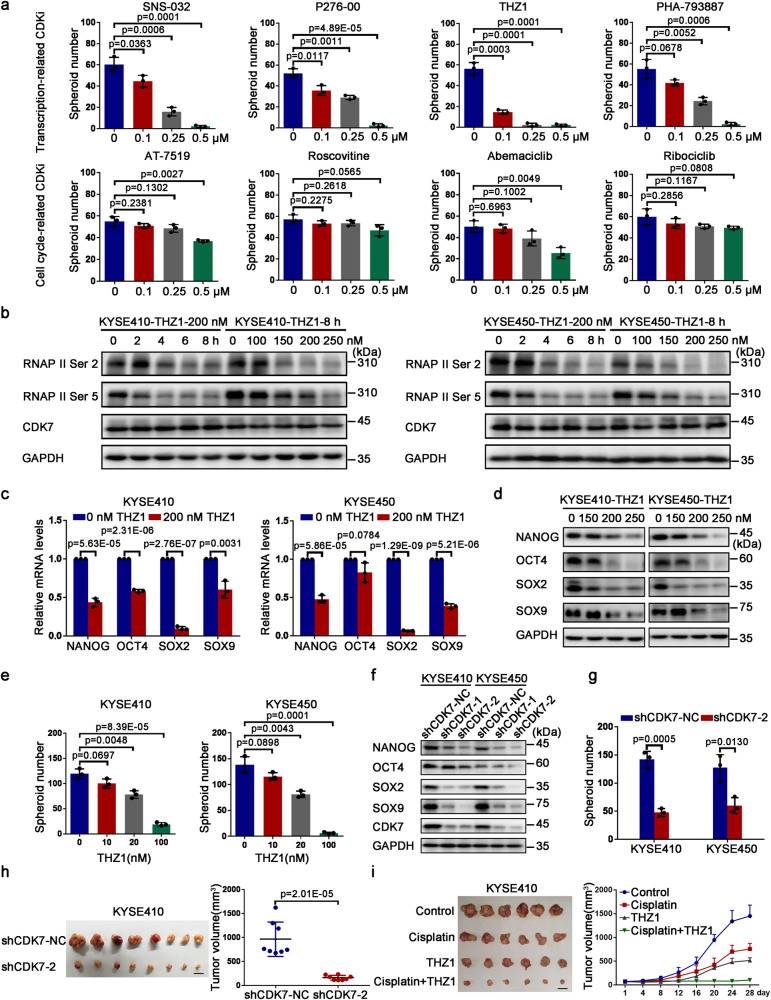
Decreased CDK7 expression leads to attenuated stem-like properties in ESCC CSCs. **a** The number of KYSE410 spheres cultured with different small molecule inhibitors was counted and graphed. **b** Immunoblotting analysis of RNAPII C-terminal domain (CTD) phosphorylation (RNAPII S2, RNAPII S5) in KYSE410 (left) and KYSE450 (right) cells treated with THZ1 at the indicated time or with the indicated concentrations. **c** KYSE410 (left) and KYSE450 (right) cells were treated with 200 nM THZ1 or DMSO for 4 h. Then total RNA was extracted and mRNA expression of *NANOG*, *OCT4*, *SOX2*, and *SOX9* were measured by qRT-PCR. **d** Immunoblotting analysis of NANOG, OCT4, SOX2, and SOX9 protein levels in KYSE410 and KYSE450 cells treated with either THZ1 or DMSO at indicated concentrations for 36 h. **e** The number of spheres from KYSE410 (left) and KYSE450 (right) cells following indicated conditions was quantified and graphed. **f** The expression of NANOG, OCT4, SOX2, and SOX9 proteins was detected by Western blot in shCDK7-transfected (shCDK7-1, shCDK7-2) cells and corresponding control (shCDK7-NC) cells. **g** The number of spheres from shCDK7-transfected (shCDK7-2) and control (shCDK7-NC) KYSE410 and KYSE450 cells was quantified and graphed. **h** shCDK7-transfected (shCDK7-2) and control (shCDK7-NC) KYSE410 cells were injected subcutaneously in BALB/C nude mice. Tumors were dissected and photographed after 4 weeks (left). Tumors were measured and graphed (right). **i** Mice bearing KYSE410 cells were treated with THZ1 (10 mg/kg), cisplatin (5 mg/kg), the combination of THZ1 and cisplatin or 0.9% NaCl. Tumors were dissected and photographed (left). Tumor growth curves of mice bearing KYSE410 cells (right). Scale bars in (**h**) and (**i**), 1 cm. GAPDH was used as an internal reference in (**b**), (**d**), and (**f**). Error bars in aforementioned histograms represent the mean ± S.D. of three independent experiments (*n* = 3)

To determine the dependency of cell cycle-related kinases in the stemness maintenance of ESCC-CSCs, we used small molecule inhibitors to repress these kinases pharmaceutically and evaluated their spheroid-formation capacity. To this end, 24 small-molecule suppressors of cell cycle-related kinases that almost all are currently under phase I and II clinical trials, involving in those for cell cycle and transcription-associated CDKs were employed to elucidate their anti-CSCs activities. KYSE410 cells were treated with these small molecules and cell viability was measured via MTS test at 72 h (Table 1). Next, their anti-esophageal CSCs potency was assessed with the help of the spheroid-formation test. It was revealed that the novel covalent CDK7 suppressor THZ1, indicated a powerful anti-ESCC-CSCs effect as reflected by the observation that the spheroid-forming potential of KYSE410 cells was suppressed dramatically after THZ1 treatment (Fig. 1a and Supplementary Fig. 1h). These results strongly demonstrate that CDK7 might be a critical molecule for the stemness maintenance of ESCC-CSCs. The previous study using transcriptome technology (RNA-Seq) indicated that a group of transcripts were specifically inhibited with low THZ1 dose, which partially suppressed RNAPII function. These transcripts were called “THZ1-sensitive transcripts” and they essentially identified cancer cell.^28^ To elucidate if the stemness-related genes expression was sensitive to THZ1 therapy, the levels of RNAPII CTD phosphorylation (S2 and S5) were examined in ESCC cells such as KYSE410 and KYSE450, which revealed that RNAPII was partially suppressed under four-hour treatment of 200 nM THZ1 (Fig. 1b). The mRNA and protein expression of *SOX9*, *SOX2*, *OCT4*, and *NANOG* notably reduced with RNAPII inhibition in these ESCC cells (Fig. 1c, d), suggesting that stemness-related genes expression was sensitive to THZ1 therapy. Additionally, THZ1 treatment could effectively suppress the sphere formation of indicated ESCC cells in a dose-dependent pattern (Fig. 1e and Supplementary Fig. 2a). Furthermore, THZ1 also markedly reduced cell growth and arrested ESCC cells in G_2_/M phase (Supplementary Fig. 2b–d). Taken together, these results revealed that CDK7 was required for ESCC-CSCs and THZ1 might be promising for targeting ESCC-CSCs.

**Table 1:** IC50 values of 24 small molecule inhibitors targeted cell cycle-related proteins

Inhibitor	Target	IC50(μM)
Rigosertib	PLK	0.407
BI 2536	PLK	>10
GSK461364	PLK	>10
BI 6727	PLK	8.02
AZD7762	ChK	2.81
LY2603618	ChK	7.072
SNS-032	CDK2/7/9	1.249
PHA-793887	CDK2/5/7	5.491
P276-00	CDK1/4/9	0.7547
Alvocidib	CDK1/2/4/6/7	0.5529
AZD5438	CDK1/2/9	4.117
Flavopiridol	CDK1/2/4/6/7	0.5095
Dinaciclib	CDK1/2/5/9	0.2828
Milciclib	CDK1/2/4/5/7	0.1711
THZ1	CDK7	0.1191
AT7519	CDK1/2/4/6	3.16
Roscovitine	CDK1/2/5	>10
Abemaciclib	CDK4/6	3.169
Ribociclib	CDK4/6	>10
BMS-863233	cdc7	>10
MLN8054	Aurora Kinase	>10
Alisertib	Aurora Kinase	>10
TAK-901	Aurora Kinase	4.896
MK-5108	Aurora Kinase	19.23

### CDK7 governs ESCC-CSCs hallmarks independently of its role in cell cycle regulation

To further determine if CDK7 participated in regulating esophageal CSCs hallmarks, CDK7 expression in indicated ESCC cells was depleted. As the data shown in Fig. 1f, the protein expression of stemness-related genes declined after CDK7 knockdown. We also observed that the self-renewal ability and migration capability were distinctly attenuated in CDK7-depleted ESCC cells (Fig. 1g and Supplementary Fig. 2e, f). To detect the effect of CDK7 on chemotherapy, we treated CDK7-knocked down and control ESCC cells with cisplatin, a first-line treatment for clinically terminal ESCC. The apoptotic rate in CDK7-knocked down cells was notably increased compared with the control cells (Supplementary Fig. 2g). We then subcutaneously inoculated CDK7-depleted and control KYSE410 cells into BALB/C nude mice. CDK7 depletion greatly inhibited proliferation potential of KYSE410 cells (Fig. 1h). To further elucidate if THZ1 therapy could increase the mortality by cisplatin in tumors, the mice harboring KYSE410 cells were given cisplatin, THZ1, or co-therapy, and the control received 0.9% NaCl. It was indicated that in THZ1 combined with cisplatin treated xenografts, the tumor volumes were significantly reduced than those which received monotherapy of cisplatin or THZ1 (Fig. 1i).

To elucidate if CSCs properties modulated by CDK7 relied on its cell cycle-regulated activity, Nocodazole was introduced in ESCC cells including KYSE410 and KYSE450, ultimately causing G_2_/M arrest. Consistently, an increased number of these ESCC cells were observed in G_2_/M phase (Supplementary Fig. 2h, i). However, the quantity of CD90 positive subset and stemness-related protein levels did not reduce after Nocodazole treatment (Supplementary Fig. 2j–l), suggesting that CDK7 modulates CSCs functions possibly via a different mechanism from its cell cycle regulatory role.

### CDK7 phosphorylates nuclear YAP at its S127 and S397 sites and induces its protein expression independently of the Hippo pathway

**Fig. 2:**
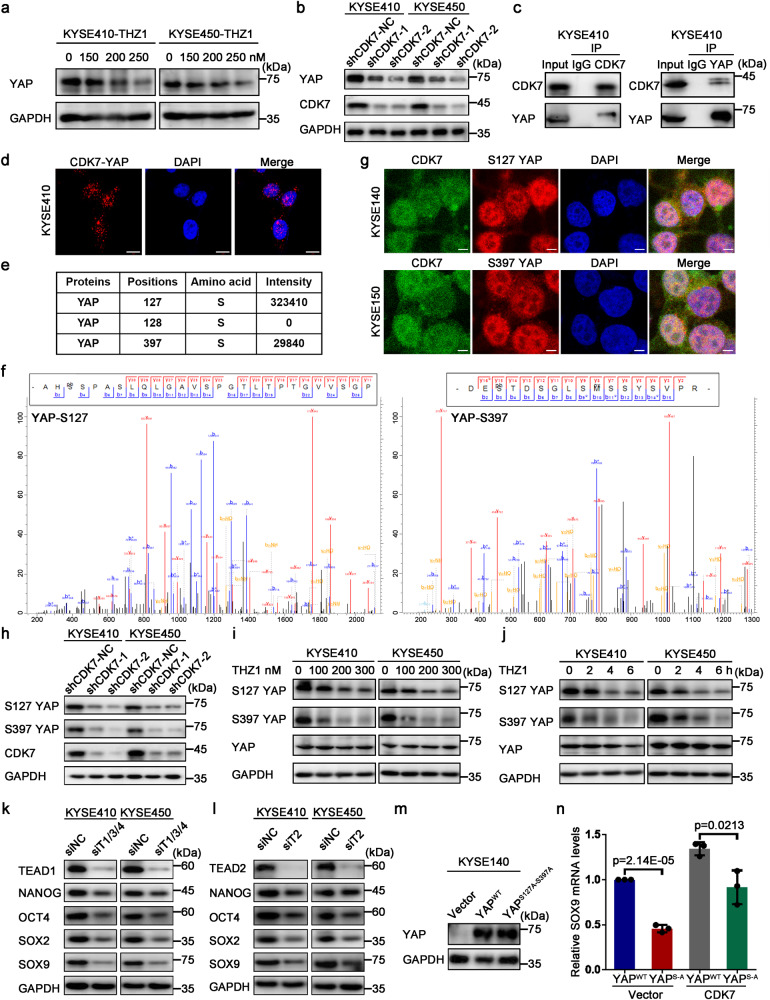
CDK7 phosphorylates YAP at S127 and S397 in the nucleus to enhance its transcriptional activity. **a** Western blot analysis of YAP protein expression in KYSE410 (left) and KYSE450 (right) cells cultured in the RPMI-1640 medium containing indicated concentration of THZ1 for 36 h. **b** Western blot analysis of YAP protein expression in shCDK7-transfected (shCDK7-1, shCDK7-2) and control (shCDK7-NC) KYSE410 and KYSE450 cells. **c** Interaction of endogenous YAP and CDK7 in KYSE410 cells. **d** Proximity ligation assay (PLA) showing the direct interaction between CDK7 and YAP. The nuclei were stained with DAPI. Scale bar, 10 μm. **e** Kinase assay in vitro suggesting the phosphorylated site and intensity of YAP mediated by CDK7. **f** Mass spectrometry peak photographs showing the phosphorylation of YAP S127 and S397 sites. **g** Immunofluorescent images representing the nuclear/cytoplasmic localization of CDK7 (Alexa Fluor 488, green) and phosphorylated YAP (S127 YAP, S397 YAP) (Alexa Fluor 543, red) in CDK7 overexpressed KYSE140 and KYSE150 cells, DAPI (blue) was used for the nuclear stain. Scale bar, 5 μm. **h** Phosphorylated YAP protein (S127, S397) levels in shCDK7-transfected (shCDK7-1, shCDK7-2) and control (shCDK7-NC) KYSE410 and KYSE450 cells were detected by Western blot. **i** Phosphorylated YAP protein (S127 YAP, S397 YAP) levels in KYSE410 and KYSE450 cells were detected by Western blot after treatment with the indicated concentrations of THZ1 for 4 h. **j** Phosphorylated YAP protein (S127 YAP, S397 YAP) levels in KYSE410 and KYSE450 cells were detected by Western blot after treatment with 200 nM THZ1 for indicated time. **k** Immunoblotting analysis of stemness-associated genes expression when TEAD1, TEAD3, TEAD4 (TEAD1/3/4) were knocked down by siRNA in KYSE410 and KYSE450 cells. **l** Immunoblotting analysis of stemness-associated genes expression when TEAD2 was knocked down by siRNA in KYSE410 and KYSE450 cells. **m** Western blot analysis of YAP protein status in wild type and transfected with lentivirus containing an empty vector or YAP^S127A-S397A^ mutant. **n** Real-time PCR analysis of SOX9 mRNA levels in CDK7-overexpressed and control KYSE140 cells when both S127 and S397 sites were mutated into alanine. GAPDH was used as an internal reference for all above-mentioned Western blot assays. Error bars represent mean ± SD (*n* = 3)

Recent findings have indicated that YAP expression was strongly determined by activated transcription, and an obviously attenuated YAP level was observed when repressing CDK7 using minor THZ1 in ESCC.^28^ To elucidate if CDK7 interacts with YAP in ESCC cells, CDK7 activity was inhibited by THZ1 treatment, and then YAP levels were assessed. THZ1 efficiently suppressed YAP expression in a time and dose-dependent pattern in ESCC cells involving in KYSE410 and KYSE450 (Fig. 2a and Supplementary Fig. 3a). Similarly, CDK7 knockdown also reduced YAP status in these cells (Fig. 2b). Subsequently, co-immunoprecipitation measurement against KYSE410 cells’ endogenous protein was carried out to assess whether CDK7 communicated with YAP, and as the data shown the presence of CDK7-YAP immunocomplexes (Fig. 2c). Besides, we observed that they could colocalize in the nucleus by an immunofluorescence assay (Supplementary Fig. 3b). Moreover, the proximity ligation assay (PLA) also confirmed the direct interaction of CDK7 and YAP (Fig. 2d). Since CDK7 is a cyclin-dependent kinase that plays its catalytic role to help transport the phosphate groups from ATP to their targeted substrates, it was hypothesized that YAP might be phosphorylated by CDK7 when they interacted. To verify our hypothesis, we performed the kinase assay in vitro by mass spectrometric detection. As expected, CDK7 was able to phosphorylate YAP at S127 and S397 sites according to mass spectrometry data, but no phosphorylated modification was detected at S128^29^ residue of YAP (Fig. 2e, f). Immunofluorescent analysis also suggested that phosphorylated YAP-S127 and YAP-S397 could colocalize with nuclear CDK7 (Fig. 2g). Interestingly, we noted that CDK7 depletion contributed to reduced phosphorylation of YAP in both KYSE410 and KYSE450 ESCC cells (Fig. 2h). To eliminate the probability that the identified difference in the levels of YAP phosphorylation was because of alterations in the levels of total YAP protein, we used THZ1 to treat KYSE410 and KYSE450 cells and found that YAP protein expression levels remained unchanged. However, a dose and time-dependent reduction in YAP (S127 and S397) phosphorylation was identified (Fig. 2i, j). Summarily, these findings suggest that CDK7 interacts with nuclear YAP in ESCC cells and subsequently phosphorylated YAP.

Because YAP functions chiefly through regulating transcription, it was assessed if CDK7 could affect YAP-induced transcription. The data suggested that CDK7 knockdown reduced *CTGF* and *CYR61* mRNA expression (Supplementary Fig. 3c), which are well-defined YAP-targeted genes.^30^ To figure out whether the decreased expression of CTGF and CYR61 in CDK7 knockdown cells is dependent on YAP, we treated YAP-depleted HEK293A cells with THZ1 and the expression of CTGF and CYR61 was rescued drastically (Supplementary Fig. 3d), which suggested the dependence of reduced CTGF and CYR61 status mediated by depleted CDK7 on YAP. As a transcriptional co-activator, YAP regulates transcription mainly through interacting with DNA-binding transcription factors (TEAD1-4) because of the lack of a DNA-binding domain.^31^ To elucidate if YAP-induced transcriptional function was crucial for stemness-related genes expression, TEAD1-4 were knocked down with their specific siRNAs in indicated ESCC cells, which reduced the protein expression of YAP targeted genes, including *SOX9*, *SOX2*, *OCT4*, and *NANOG* (Fig. 2k, l). Moreover, the reduced expression of these stemness-related genes induced by THZ1 treatment depended on YAP-mediated transcription activity (Supplementary Fig 3e, f). In order to figure out whether YAP’s S127 and S397 sites were critical for CDK7-mediated function, we detected *SOX9* mRNA levels when both S127 and S397 sites were mutated into alanine in CDK7-overexpressed and control KYSE140 cells, and results indicated that the mutation was less responsive to CDK7-mediated potentiation (Fig. 2m, n). In summary, these data illustrate that CDK7 could govern YAP-mediated transcriptional activity.

The literature suggests that YAP protein levels are significantly modulated via LATS1 and LATS2, which are canonical Hippo signaling pathway upstream kinases.^32^ Consistently, knocking down LATS1 or LATS2 with siRNA markedly reduced the levels of phosphorylated YAP S127 and S397, and accordingly increased the total protein levels of YAP in the KYSE410 and KYSE450 cells (Supplementary Fig. 3g, h). To evaluate if CDK7 modulated YAP protein expression via the Hippo pathway, CDK7 knockdown was performed, which suggested that LATS1, MST1, and phosphorylated LATS1/2 protein expression remained unaltered (Supplementary Fig. 3i). Similar data were acquired after CDK7 overexpression (Supplementary Fig. 3j). Collectively, these data reveal that CDK7 regulates YAP expression in a Hippo pathway-independent manner.

### YAP is essential to acquire and maintain stem-like properties in ESCC cells

**Fig. 3:**
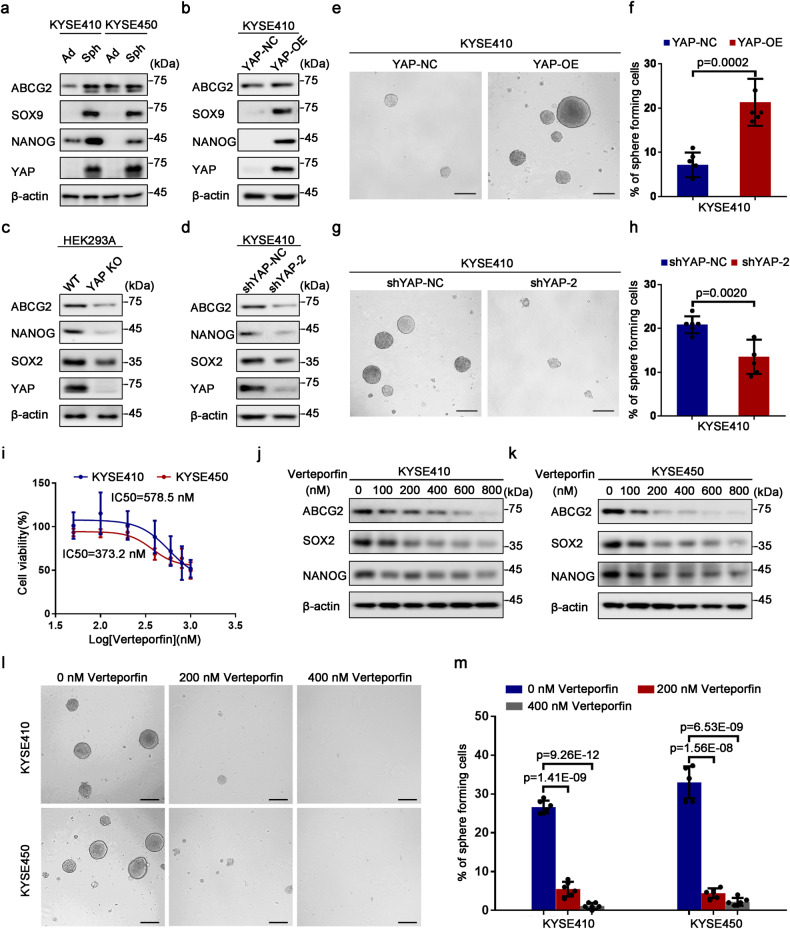
The role of YAP in the acquisition and maintenance of ESCC CSCs properties. **a** Western blotting analysis for the expression of YAP and stemness-related indicators including ABCG2, SOX9 and NANOG in adherent (Ad) and spheres-forming (Sph) KYSE410 and KYSE450 cells. **b** Immunoblotting measurement showing the change of these stemness-associated indicators statuses in YAP-overexpressed and control KYSE410 cells. **c**, **d** Western blot analysis to test the protein expression of ABCG2, NANOG and SOX2 in YAP-knocked out HEK293A and YAP-knocked down KYSE410 cells, as well as their control cells. **e** Representative photographs showing spheroids formed by KYSE410 cells after overexpressing YAP. **f** Histogram displaying the effect of forced YAP on sphere-forming ability of KYSE410 cells. **g** Representative phase contrast micrographs demonstrating spheres formation after repressing YAP in KYSE410 cells. **h** Histogram exhibiting the impact of decreased YAP by specific shRNAs on spheroids formation efficiency. **i** MTS assay showing the IC50 value of verteporfin in KYSE410 and KYSE450 cells. **j**, **k** Immunoblot analysis to detect the protein levels of stemness-indicated molecules in KYSE410 and KYSE450 cells treated with indicated concentration of verteporfin for 72 h. **l** Representative graphs of spheres formation in KYSE410 and KYSE450 cells treated with verteporfin at the indicated concentration. **m** Histogram suggesting alternative spheroids-forming efficiency by suppressing YAP activity with indicated concentration of verteporfin. Error bars in (**f**), (**h**) and (**m**) represent the mean ± S.D. of three independent experiments (*n* = 6). Scale bars in (**e**), (**g**) and (**l**) represent 100 μm. β-actin was used as an internal reference for Western blot analysis

Considering the aforementioned phenomena, it was hypothesized that YAP might substantially regulate ESCC-CSCs stemness. Consistent with our hypothesis, we found that stemness-related genes involving in *ABCG2*, *SOX9* and *NANOG* were enriched in KYSE410 and KYSE450 spheroids compared to adherent ESCC cells, as well as YAP, which has a higher expression status in ESCC stem-like cells than the differentiated ESCC cells (Fig. 3a). To test whether YAP was necessary for ESCC-CSCs, we overexpressed YAP in KYSE410 cells. As we expected, these stem cell-like indicator molecules were upregulated obviously followed by enhancing expression of YAP (Fig. 3b). For loss of function assays, we hardly observed the protein expression of stemness-associated genes when knocked-out YAP in HEK293A cells (Fig. 3c). Similarly, we discovered that these indicated molecules were suppressed significantly after YAP knockdown. (Fig. 3d). We also performed a spheroid-formation assay in vitro to assess the impact of YAP on the self-renewal potential of ESCC CSCs, as the data demonstrated that the sphere number of YAP-overexpressed KYSE410 cells was increased dramatically relative to these control cells (Fig. 3e, f). Otherwise, the decreased YAP expression resulted in a reduced sphere-forming capacity distinctly in KYSE410 cells (Fig. 3g, h).

To further confirm that YAP was essential to maintain stem-like properties of ESCC CSCs, we treated KYSE410 and KYSE450 cells with Verteporfin, a small molecule inhibitor of YAP, which mainly suppresses the interaction between YAP and TEAD. We added Verteporfin to these cells and performed MTS assay after 72 h, as the result shown that the IC50 value of Verteporfin was 578.5 nM and 373.2 nM in KYSE410 and KYSE450 cells (Fig. 3i). Next, we used various doses of Verteporfin to treat ESCC cells for 72 h and found that the protein status of stemness-related molecules involving ABCG2, SOX2 and NANOG presented a downward trend following the increasing Verteporfin concentrations (Fig. 3j, k). Similarly, the sphere-forming efficiencies were evidently decreased in KYSE410 and KYSE450 cells treated with Verteporfin (Fig. 3l, m). In summary, all above-described data illustrate that YAP was responsible for acquiring and maintaining stem cell-like characteristics of ESCC CSCs.

### LDHD serves as a key target protein driven by CDK7-YAP axis

**Fig. 4:**
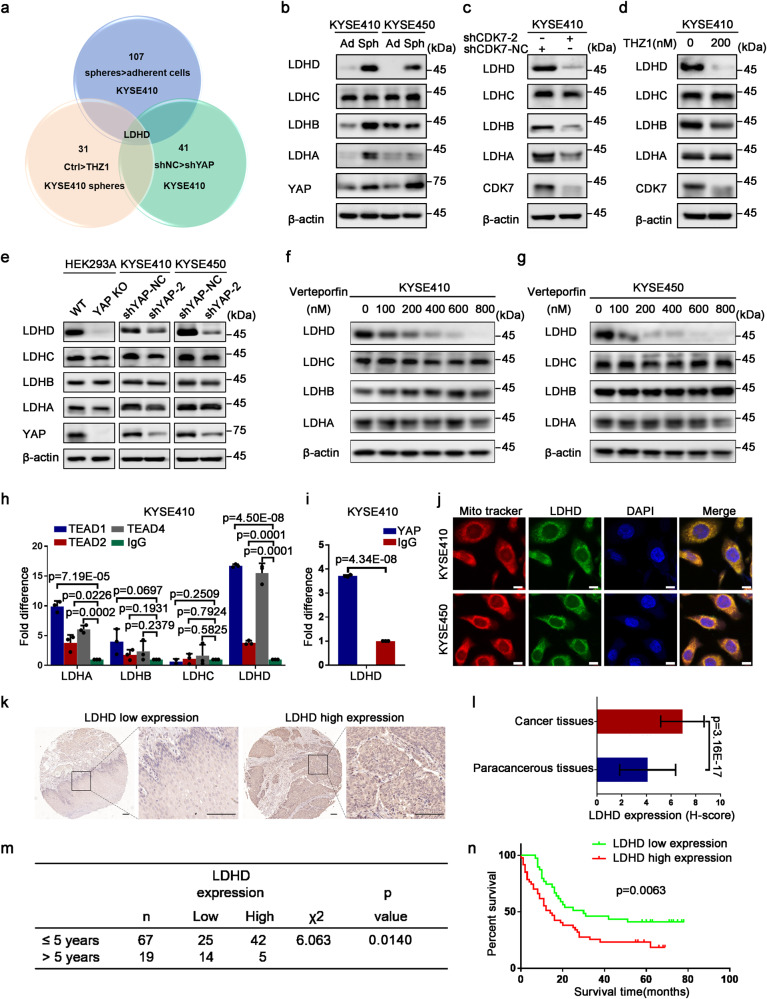
CDK7-YAP complex drives the expression of mitochondrial LDHD protein. **a** Venn diagram showing a mode for screening downstream targeted molecule of CDK7-YAP axis. **b** Western blot result demonstrating the protein status of LDH family members and YAP in adherent (Ad) cells and spheres (Sph) formed by KYSE410 and KYSE450 cells. **c**, **d** Immunoblotting analysis to measure the expression of LDH family after inhibiting CDK7 with specific shRNAs or 200 nM THZ1 treatment for 36 h. **e** Western blot analysis suggesting the changes of LDH family protein level in YAP-knocked out HEK293A and YAP-knocked down KYSE410 and KYSE450 cells. **f**, **g** Western blotting analysis for testing protein expression of LDH family treated with verteporfin at the indicated concentration for 72 h. **h** Real time PCR measurement on ChIP illustrating the combining capacity between TEAD and LDH family members. **i** ChIP-qPCR analysis showing the combination ability of YAP and LDHD. **j** Immunofluorescent staining of LDHD and mito-tracker displaying their co-location in KYSE410 and KYSE450 cells. Nuclei were stained with DAPI. Scale bars represent 5 μm. **k** Immunohistochemical staining of LDHD showing its protein expression in ESCC tissues and corresponding normal tissues. Scale bars represent 600 μm and 200 μm, respectively. **l** Histogram displaying the altered expression of LDHD protein in 86 pairs of ESCC tissues and corresponding normal tissues. **m**, **n** Chi-square test and Kaplan-Meier curves analysis suggesting the correlation between ESCC patients’ survival time and LDHD protein expression. β-actin was used as an internal reference in (**b**–**g**). Data in (**h**) and (**i**) were presented as mean ± S.D (*n* = 3)

As we all know that YAP plays a crucial role in the diverse metabolic pathways, meanwhile YAP also serves as an effector of multiple metabolic factors. Therefore, we put forward a hypothesis that metabolic reprogramming driven by CDK7-YAP axis leads to the acquisition and maintenance of ESCC-CSCs. To verify the hypothesis, we analyzed 2752 metabolic genes, including human metabolic enzymes and small molecule transporters.^33^ Among them, 107 genes were upregulated in KYSE410 spheres than adherent cells (Spheroid-related gene set_1), 31 genes were down-regulated after treatment with THZ1 (THZ1-related gene set_2), and 41 genes expression were reduced when YAP was knocked down (YAP-related gene set_3). Interestingly, there was only one gene *LDHD* shared in these three gene sets, suggesting that CDK7/YAP might coordinatively regulate LDHD expression in ESCC CSCs (Fig. 4a). According to the RNA-seq data from YAP-depleted and control KYSE410 cells, we found 185 up-regulated and 413 down-regulated genes when YAP was knocked down (Supplementary Fig. 4a). The mRNA levels of LDH family members changed dramatically, especially for LDHD (Supplementary Fig. 4b), meanwhile multiple metabolic pathways changed significantly including oxidative phosphorylation (Supplementary Fig. 4c). After the expression of CDK7 was suppressed by shRNA targeted CDK7 or treated with THZ1, the LDH family members’ mRNA levels were down-regulated and among them, LDHD was the most significant one (Supplementary Fig. 4d, e). Similarly, LDHD mRNA expression was reduced dramatically when interfered YAP expression in KYSE410 and KYSE450 cells (Supplementary Fig. 4f, g). We subsequently tested the protein status of LDH family in adherent cells and spheroids from KYSE410 and KYSE450, Western blot analysis showed that LDHD had the highest enrichment in ESCC spheroids compared to adherent cells (Fig. 4b). When CDK7 expression was suppressed using shRNA or THZ1 treatment for 36 h, the protein levels of LDH family were reduced and such alteration for LDHD was the most obvious (Fig. 4c, d). Consistently, the enrichment of LDHD protein was down-regulated after YAP was eliminated by sgRNA or shRNA in HEK293A and ESCC cell lines (Fig. 4e). It was also shown that LDHD decreased continuously under the action of different doses of Verteporfin in KYSE410 and KYSE450 cells, but no apparent changes for LDHA, LDHB and LDHC (Fig. 4f, g). Moreover, we performed ChIP assay to verify the combination between TEAD and LDH family members. As the data indicated that LDHD had the strongest combining ability to TEAD among four LDH family members (Fig. 4h), further combined with YAP (Fig. 4i). We also confirmed the interaction between LDHD and YAP-TEAD complex by co-immunoprecipitation analysis (Supplementary Fig. 4h, i). Additionally, we observed that THZ1 treatment for 36 h could not result in less LDHD expression when YAP was knocked out in HEK293A cells (Supplementary Fig. 4j). Therefore, YAP may positively regulate LDHD expression and LDHD serves as a downstream molecule of CDK7-YAP axis.

It has been reported that LDHD localizes in the mitochondria.^34^ To confirm the location of LDHD in KYSE410 and KYSE450 cells, we firstly stained these cells with mito-tracker and FITC-labeled LDHD antibody simultaneously and the immunofluorescent staining photography demonstrated that LDHD had a very clear location with cellular mitochondria in ESCC cells (Fig. 4j). Besides, we also detected the apparent enrichment of LDHD protein in ESCC cells’ mitochondrion (Supplementary Fig 4k). To probe the expression of LDHD in ESCC patients, we performed immunohistochemistry and found that LDHD had a higher enrichment in ESCC tumor tissues than in paired adjacent tissues (Fig. 4k, l). Then we analyzed the correlation between LDHD protein status and survival time of 86 ESCC patients, and observed that ESCC patients with high LDHD levels had a reduced survival time (Fig. 4m, n), suggesting that LDHD might serve as an oncogenic protein and responds to CDK7-YAP axis.

### LDHD functions as a determinant in the regulation of ESCC cells growth and metastasis

**Fig. 5:**
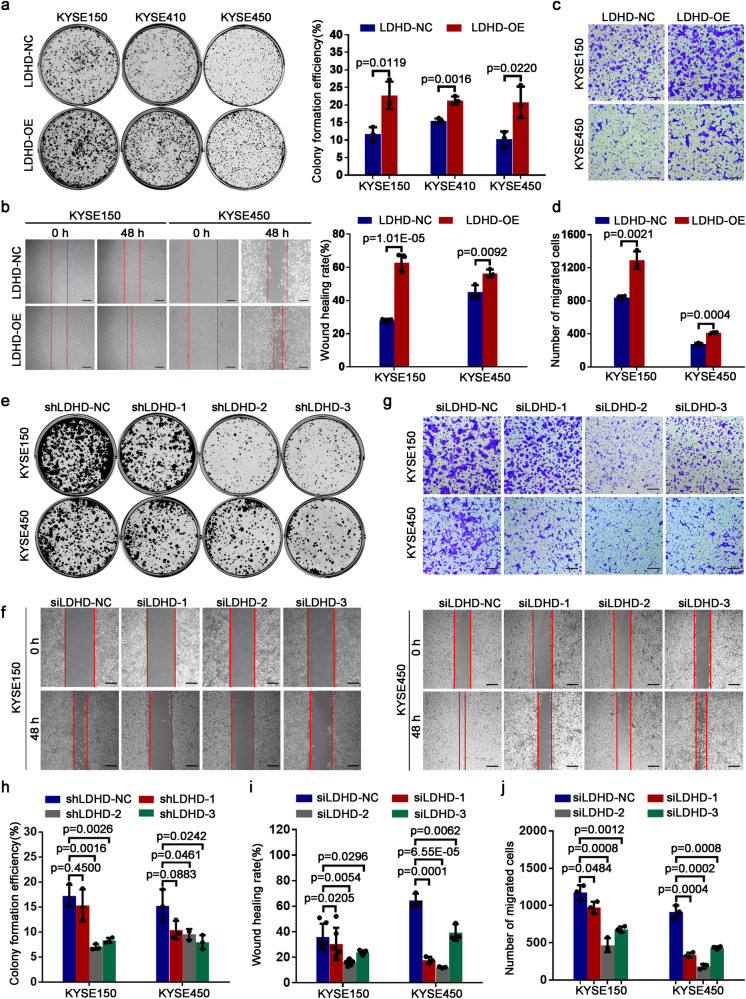
LDHD is responsible for tumor cells proliferation and migration in ESCC. **a** Representative graph showing the colonies formed by increased LDHD in KYSE150, KYSE410 and KYSE450 cells (left) and the histogram displaying the colonies-forming efficiencies (right). **b** Scratch wound healing assay results suggesting the impact of forced LDHD expression on the wound healing ability in KYSE150 and KYSE450 cells (left) and the histogram showing the wound healing rate of these cells with an increasing LDHD protein (right). **c** Transwell assay analysis displaying an alternative migrated capacity of LDHD overexpressing KYSE150 and KYSE450 cells and (**d**) the histogram showing their migrated efficiencies. **e** Colony formation assay demonstrating the changes of proliferative potential in KYSE150 and KYSE450 cells following attenuated LDHD protein by specific shRNAs. **f** Representative phase contrast micrographs indicating the wound healing ability of KYSE150 (left) and KYSE450 (right) cells with decreased LDHD protein status with their siRNAs. **g** Transwell assay for the measurement of migrated ability of KYSE150 and KYSE450 cells after repressing LDHD protein levels. **h** Histogram showing the colony formation efficiency in LDHD-depleted KYSE150 and KYSE450 cells. **i** Histogram exhibiting the scratch wound healing rate in KYSE150 and KYSE450 cells when interfering LDHD expression by specific siRNAs. **j** Histogram suggesting the numbers of migrated cells when LDHD was knocked down. Scale bars represent 100 μm. Data were presented as mean ± S.D (*n* = 3)

Given the investigations of LDHD are limited, the function of LDHD in the regulation of tumor initiation and progression remains unclear. To explore the role of LDHD during ESCC tumorigenesis, we first performed a series of malignant phenotypes-associated assays. Increasing efficiencies of colony formation were observed in all three LDHD overexpressed ESCC cells (Fig. 5a). The cell cycle assessment also revealed that forced LDHD expression enhanced the ratio of cells distributed in S phase and promoted the proliferation of ESCC cells (Supplementary Fig. 5a, b). Additionally, high expression of LDHD in KYSE150 and KYSE450 largely increased wound healing rate (Fig. 5b) and demonstrated a stronger migrated ability (Fig. 5c, d).

We next knocked down LDHD expression in KYSE150 and KYSE450 cells and performed colony formation assay and cell cycle analysis. Clearly, decreased LDHD led to a reduced colony-forming rate (Fig. 5e, h) and less distribution of ESCC cells in S phase (Supplementary Fig. 5c–e). As expected, decreased wound healing efficiency (Fig. 5f, i) and lower migrated rate (Fig. 5g, j) were observed in the cells with silenced LDHD. Collectively, LDHD is a strong oncogenic molecule during malignant progression in ESCC cells.

### LDHD is responsible for stemness-associated hallmarks of ESCC cells

**Fig. 6:**
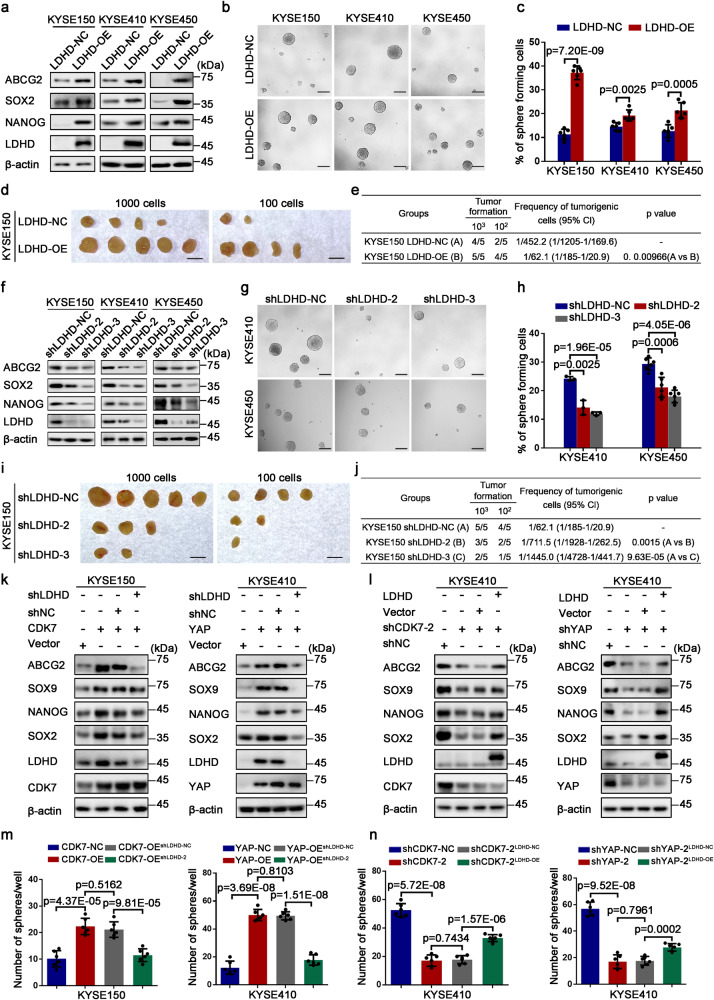
CDK7-YAP-LDHD axis drives stemness-associated hallmarks of ESCC CSCs. **a** Western blotting analysis for detecting the expression of stemness-associated indicators in LDHD-overexpressed KYSE150, KYSE410 and KYSE450 cells. **b** Representative images demonstrating the spheres formed by KYSE150, KYSE410 and KYSE450 cells infected with empty vector and LDHD-overexpressed lentivirus. **c** Histogram displaying the impact of forced LDHD on sphere formation efficiency of these cells. **d** Photographs showing the dissected tumors formed by KYSE150 cells after LDHD overexpression in NOD/SCID mice. **e** The tumor-initiating frequencies of LDHD-overexpressed and control KYSE150 cells. **f** Immunoblotting analysis for the expression of indicated molecules in KYSE150, KYSE410 and KYSE450 cells after knocking down the expression of LDHD with shRNAs. **g** Representative photographs demonstrating the spheroids formed by KYSE410 and KYSE450 cells following suppressing LDHD expression by specific shRNAs. **h** Histogram illustrating the effect of reduced LDHD protein status on spheroid formation ability. **i** Images suggesting the dissected tumors formed by KYSE150 cells after knocking down LDHD. **j** The tumor-initiating frequencies of LDHD-depleted and control KYSE150 cells. **k** Western blotting results exhibiting the alternative protein levels of indicted molecules after knocking down LDHD in CDK7-overexpressed KYSE150 (left) and YAP-overexpressed KYSE410 (right) cells. **l** Western blot analysis for the expression of indicated molecules in CDK7-knocked down (left) and YAP-knocked down (right) KYSE410 cells after overexpressing LDHD. **m** Histograms showing the changing sphere-forming efficiencies of CDK7-overexpressed KYSE150 (left) and YAP-overexpressed (right) KYSE410 cells following knocking-down LDHD. **n** Histograms indicating the impact of forced LDHD expression on the spheroids formation efficiencies in CDK7-knocked down (left) and YAP-knocked down (right) KYSE410 cells. β-actin was used as an internal reference in Western blot experiments. Data were presented as mean ± SD of three independent experiments (*n* = 6). Scale bars in (**b**) and (**g**) represent 100 μm, 1 cm for (**d**) and (**i**)

Since LDHD had a higher enrichment in ESCC CSCs than differentiated cells, we subsequently explored the effect of LDHD on stemness-related properties for ESCC-CSCs and observed that these stem cell-like indicator molecules such as ABCG2, SOX2 and NANOG were up-regulated dramatically in all three LDHD overexpressed ESCC cells (Fig. 6a). The sphere-forming potential of these cells was also elevated apparently following high expression of LDHD (Fig. 6b, c). To evaluate the impact of LDHD on tumor-forming capacity in vivo, we transplanted subcutaneously 1000 and 100 LDHD-overexpressed and control KYSE150 cells per site into NOD/SCID mice, then we found that increasing LDHD greatly contributed to a stronger tumorigenic potential than control group as reflected by increased frequency of tumorigenic cells (Fig. 6d, e). Therefore, LDHD expression enhances tumorigenicity of esophageal CSCs.

Consistently, the reduced expressions of stemness-related indicator molecules were observed in these ESCC cell lines with depleted LDHD (Fig. 6f). The spheres-forming capacity was also reduced significantly following LDHD was depleted in KYSE410 and KYSE450 cells (Fig. 6g, h). Subsequently, KYSE150 cells with a decreased LDHD expression and their control cells were transplanted subcutaneously into NOD/SCID mice at 1000 and 100 per site to examine their tumorigenicity in vivo. Compared to the control cells, the tumorigenic ability of KYSE150 cells with knocked-down LDHD was dramatically attenuated (Fig. 6i, j). Taken together, we conclude that LDHD is critically involved in the acquisition and maintenance of stemness-associated properties of ESCC CSCs.

### CDK7-YAP-LDHD axis sustains stem cell-like potential in ESCC cells

To verify the crosslinked relationship among CDK7, YAP, and LDHD in the regulation of ESCC CSCs properties, we performed a series of rescue assays. Firstly, we knocked down the expression of LDHD in CDK7-overexpressed KYSE150 and YAP-overexpressed KYSE410 cells, respectively. Surprisingly, Western blot data illustrated that increased expression of stemness-related indicators including ABCG2, SOX9, NANOG and SOX2 mediated by CDK7 and YAP was reduced remarkably after knocking down LDHD (Fig. 6k). Moreover, the reduced protein levels of these stem cell-like genes driven by down-regulated CDK7 or YAP were rescued significantly when we overexpressed LDHD in CDK7 and YAP-depleted KYSE410 cells (Fig. 6l). Consistently, attenuated self-renewal capacity was achieved in CDK7-overexpressed KYSE150 and YAP-overexpressed KYSE410 cells by interfering the expression of LDHD (Fig. 6m and Supplementary Fig. 6a), whereas decreasing sphere-forming potential mediated by reduced CDK7 or YAP was up-regulated obviously when we restored the expression of LDHD in KYSE410 cells (Fig. 6n and Supplementary Fig. 6b). In addition, increased colony formation efficiency induced by CDK7 and YAP was inhibited apparently after suppressing LDHD expression in CDK7 and YAP-overexpressed ESCC cells (Supplementary Fig. 6c, d), and increased colonies were also observed in CDK7 or YAP-knocked down KYSE410 cells following high expression of LDHD (Supplementary Fig. 6e, f). These data indicate that CDK7-YAP-LDHD axis is a key determinant for stemness-associated hallmarks in ESCC cells.

### D-lactate/pyruvate transition driven by CDK7-YAP-LDHD axis enhances ESCC-CSCs’ properties

To explore the metabolic phenotype of ESCC CSCs driven by CDK7-YAP-LDHD axis, we tested the intracellular glucose using a 100 μM 2-NBDG, which is an analog of glucose labeled with FITC and found that relative glucose uptake efficiency was down-regulated notably after suppressing the expression of CDK7 and YAP in ESCC cells (Supplementary Fig. 7a, b). Meanwhile, we collected the equal YAP/LDHD-overexpressed and their control KYSE410 cells, then performed an untargeted metabolomics analysis to seek possible metabolic pathways regulated by CDK7-YAP-LDHD axis. We found that forced YAP expression led to 592 up-regulated and 711 down-regulated metabolites in KYSE410 cells (Supplementary Fig. 7c). Consistently, 382 up-regulated and 186 down-regulated metabolites were observed in KYSE410 cells following increasing LDHD expression (Supplementary Fig. 7d), and these metabolites were involved in various metabolic pathways such as pyruvate metabolism and TCA cycle (Supplementary Fig. 7e). Besides, we also noted that several metabolic intermediates in TCA cycle and amino acid metabolism pathway were up-regulated in both YAP-overexpressed and LDHD-overexpressed KYSE410 cells by accident, such as succinate, malate and β-alanine, which can be converted into pyruvate by transdeamination (Supplementary Fig. 7g, h).

**Fig. 7:**
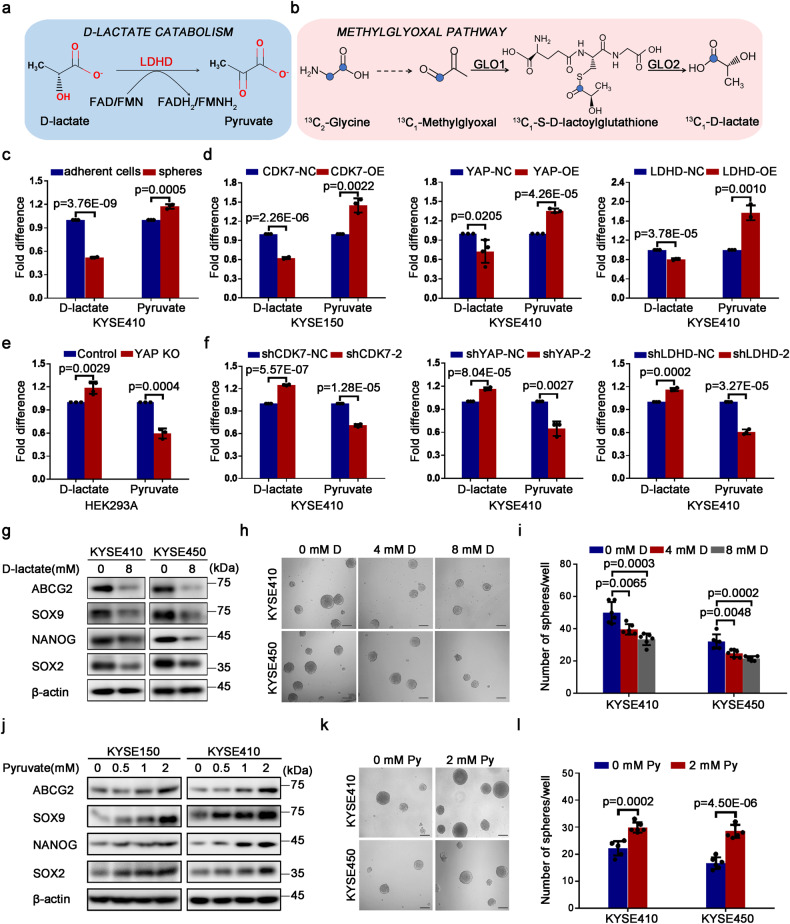
D-lactate/pyruvate transition mediated by CDK7-YAP-LDHD axis regulates ESCC CSCs properties. **a** Diagram of D-lactate catabolism in mitochondria. **b** Untargeted metabolomic mass spectrometry analysis showing methylglyoxal pathway in KYSE410 cells. **c** D-lactate and pyruvate assays for their changed content in adherent and spheroid-forming KYSE410 cells. **d** D-lactate and pyruvate analysis to measure their levels in CDK7-overexpressed KYSE150, YAP-overexpressed and LDHD-overexpressed KYSE410 cells. **e** Cellular D-lactate and pyruvate content were detected with corresponding kits in YAP-knocked out HEK293A cells. **f** D-lactate and pyruvate analysis in CDK7, YAP or LDHD knocked-down KYSE410 cells. **g** Western blot analysis for the expression of indicted molecules in KYSE410 and KYSE450 cells supplementing 8 mM D-lactate for 72 h. **h** Representative micrographs suggesting the spheres formed by KYSE410 and KYSE450 cells treated with D-lactate (D) at indicated concentration. **i** Histogram showing the numbers of spheres in KYSE410 and KYSE450 cells supplementing indicated concentration of D-lactate (D). **j** Western blotting analysis for the expression of indicated molecules in KYSE150 and KYSE410 cells treated with indicated pyruvate for 72 h. **k** Representative phase contrast micrographs displaying the effect of 2 mM pyruvate (Py) on spheroids-forming capacity in KYSE410 and KYSE450 cells. **l** Histogram illustrating their changed spheroids formation efficiency in (**k**). β-actin was used as an internal reference in (**g**) and (**j**). Scale bars in (**h**) and (**k**) represent 100 μm. Error bars represent mean ± SD, (*n* = 3) in (**c**–**f**), (*n* = 6) in (**i**) and (**l**)

Mitochondrial LDHD is the key catalytic enzyme for the catabolism of D-lactate, changed into pyruvate (Fig. 7a). Combined with the data from untargeted metabolomics analysis, we decided to focus on D-lactate catabolism in mitochondria. We analyzed the metabolic flux of D-lactate in KYSE410 cells through stable isotope experiments based on [U-^13^C_2_] glycine, which can be converted into methylglyoxal, followed by lactoylglutathione and D-lactate with one labeled carbon (Fig. 7b). According to metabolic flux analysis, we preliminarily confirmed that D-lactate could be generated in mammals. We used D-lactate assay kit and pyruvate assay kit to measure intracellular D-lactate and pyruvate of adherent cells and spheres from KYSE410, respectively. It was clearly shown that D-lactate was lower in esophageal CSCs than in differentiated cells, interestingly, a higher amount of pyruvate was detected in CSCs relative to adherent cells (Fig. 7c). Similarly, we found that D-lactate was reduced and pyruvate was elevated after overexpressing CDK7, YAP and LDHD in ESCC cells (Fig. 7d). THZ1 treatment could promote D-lactate generation in a dose-dependent pattern for KYSE410 and KYSE450 cells (Supplementary Fig. 7f). Conversely, attenuated CDK7, YAP or LDHD expression resulted in an increased D-lactate and a decreased pyruvate production in KYSE410 cells, even in YAP-knocked out HEK293A cells (Fig. 7e, f). Hence, we believed that decreasing D-lactate and forced pyruvate production are required for ESCC CSCs.

What’s the specific significance of the low D-lactate and high pyruvate phenotype for esophageal CSCs? We attempted to add different concentrations of D-lactate and L-lactate to KYSE410 cells and incubated for 72 h. Unexpectedly, the stemness-associated indicators including ABCG2, SOX9, NANOG, and SOX2 were down-regulated dramatically after treatment with D-lactate, but no obvious changes for the treatment of L-lactate (Supplementary Fig. 8a, d). Moreover, increasing D-lactate could lead to a reduced esophageal spheres-forming ability in KYSE410 cells (Supplementary Fig. 8e, f), but no apparent alternations after adding L-lactate (Supplementary Fig. 8b, c). Data from colony formation assay also confirmed that 2 mM D-lactate resulted in a decreased colony-forming capacity for KYSE410 cells, but no response to L-lactate (Supplementary Fig. 8g). Hence, we speculate that excess intracellular D-lactate might affect stemness-associated properties for ESCC cells instead of equal L-lactate. To verify the hypothesis, the role of D-lactate in regulating esophageal tumorigenesis was primarily focused. We added a higher concentration of D-lactate to treat KYSE410 and KYSE450 cells for 72 h, and observed a decreased protein status of stem cell-like indicators (ABCG2, SOX9, NANOG, and SOX2) (Fig. 7g). Similarly, the esophageal spheres formation efficiency of KYSE410 and KYSE450 cells was attenuated in a dose-dependent pattern under the treatment of D-lactate (Fig. 7h, i). In order to certify whether there was a metabolic synergistic effect between decreased D-lactate accumulation and elevated production of pyruvate, we added various concentrations of pyruvate to KYSE150 and KYSE410 cells and cultured these cells for 72 h. As we expected, the expression of stem cell-like molecules (ABCG2, SOX9, NANOG, and SOX2) was enhanced significantly (Fig. 7j), also increasing sphere formation potential was achieved (Fig. 7k, l). In addition, exogenous pyruvate could result in increasing colony-forming efficiency in KYSE410 and KYSE450 cells (Supplementary Fig. 8h). Therefore, it is reasonable to suspect that pyruvate mainly serves as the substrate of TCA cycle to provide more energy for ESCC CSCs. In summary, the metabolic pattern of decreasing D-lactate and increasing pyruvate driven by CDK7-YAP-LDHD axis in mitochondria was needed for maintaining the self-renewal and proliferative potential in ESCC CSCs.

### Mitochondrial D-lactate catabolism protects ESCC cells from ferroptosis to support CSCs’ hallmarks

**Fig. 8:**
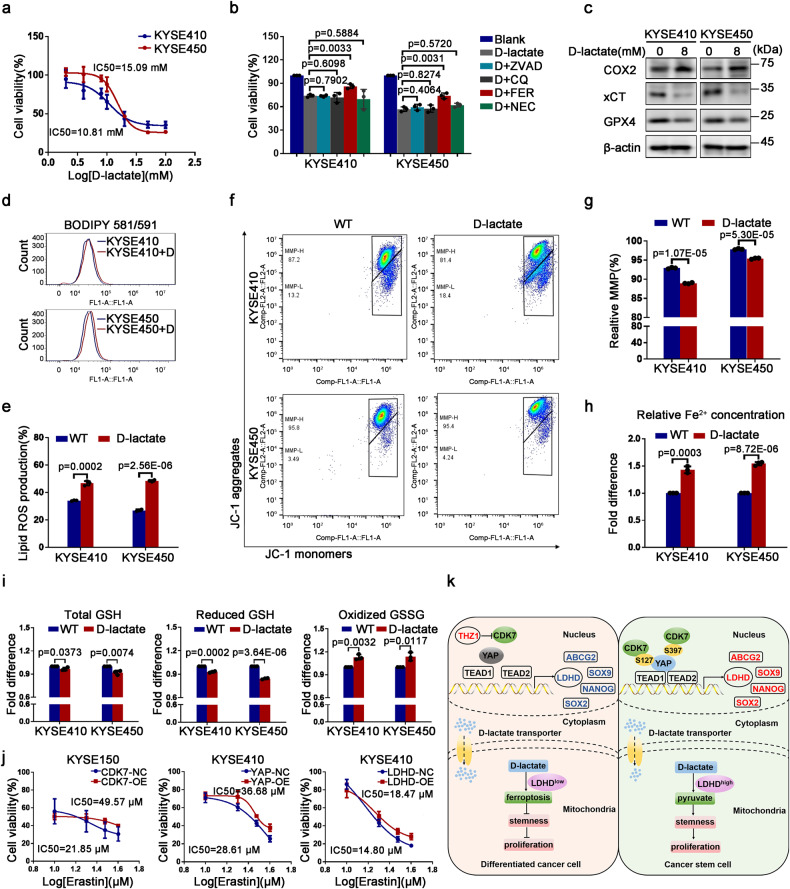
D-lactate catabolism prevents ESCC CSCs ferroptosis to sustain their stem-like functions. **a** Cell viability analysis showing the IC50 value of D-lactate in KYSE410 and KYSE450 cells. **b** MTS measurement for cell viability of KYSE410 and KYSE450 cells treated with 8 mM and 16 mM D-lactate (D) alone or supplementing ZVAD, CQ, FER and NEC simultaneously. **c** Immunoblotting results suggesting the expression of ferroptosis markers in KYSE410 and KYSE450 cells with 8 mM or 16 mM D-lactate for 72 h. β-actin was used as an internal reference. **d** Flow cytometry analysis for lipid reactive oxygen species generation in KYSE410 and KYSE450 cells treated with D-lactate (D). **e** Histogram showing the relative reactive oxygen species production under the action of 8 mM or 16 mM D-lactate in KYSE410 and KYSE450 cells, respectively. **f** Flow cytometry results indicating the alternative mitochondrial membrane potential induced by 8 mM or 16 mM D-lactate. JC-1 monomers and JC-1 aggregates represent low and high MMP, respectively. **g** Histogram demonstrating relative mitochondrial membrane potential in KYSE410 and KYSE450 cells treated with D-lactate. **h** Histogram indicating changed Fe^2+^ concentration induced by D-lactate in KYSE410 and KYSE450 cells. **i** Histograms suggesting the changes of intracellular total GSH, reduced GSH and GSSG content in KYSE410 and KYSE450 cells treated with D-lactate. **j** MTS measurement showing the sensitivity of ESCC cells on Erastin treatment after CDK7-YAP-LDHD axis was up-regulated. **k** Working model of D-lactate catabolism driven by CDK7-YAP-LDHD axis promotes pyruvate generation and helps ESCC cells escape from ferroptosis induced by D-lactate, then support ESCC-CSCs’ properties. Error bars represent mean ± SD (*n* = 3)

We were curious about how D-lactate regulates stemness-related hallmarks of ESCC CSCs. Consequently, we added 8 mM D-lactate to KYSE410 cells for 72 h and then performed RNA-seq analysis. There were 302 up-regulated and 496 down-regulated genes after D-lactate treatment and these genes were enriched in multiple signaling pathways including the pathway associated with the regulation of stem cells pluripotency (Supplementary Fig 9a, b). Furthermore, the mRNA status of SOX2 was down-regulated apparently, which was a famous stemness-related transcription factor (Supplementary Fig. 9c). Cell viability analysis showed that the IC50 value of D-lactate in KYSE410 and KYSE450 was 10.81 mM and 15.09 mM, respectively (Fig. 8a). We then added 8 mM or 16 mM D-lactate to KYSE410 and KYSE450 cells, in the presence of 20 μM ZVAD-fmk (ZVAD), 50 μM Chloroquine (CQ), 1 μM Ferrostain-1 (Fer-1), and 30 μM Necrostain-1 (Nec-1), which are corresponding to the inhibitors of cell apoptosis, autophagy, ferroptosis, and necrosis pathway. 72 h after treatment, MTS data indicated that KYSE410 and KYSE450 cells treated with Fer-1 displayed the strongest cell viability (Fig. 8b), suggesting that accumulated D-lactate could contribute to cells death in the manner of ferroptosis. Consistently, RNA-seq analysis also hinted the altered mRNA expression of ferroptosis-associated genes including *SLC7A11* and *MT-CO2*, which encoded cystine/glutamate transporter (xCT) and cytochrome C oxidase subunit 2 (COX2) protein, respectively (Supplementary Fig. 9c). We then analyzed the protein levels of xCT, COX2 and glutathione peroxidase 4 (GPX4), which are regarded as the markers when cells occurred ferroptosis. Western blotting data demonstrated that 8 mM D-lactate was able to result in increasing COX2, while attenuated GPX4 and xCT distinctly, which presented the enhancing ferroptosis (Fig. 8c). Based on the above-mentioned clues, a series of measurements about ferroptosis-associated phenotypes were performed. Firstly, we detected lipid reactive oxygen species (ROS) content with a BODIPY 581/591 and found that increased D-lactate led to elevated ROS production in KYSE410 and KYSE450 cells (Fig. 8d, e). On the other hand, elevated D-lactate resulted in a lower mitochondrial membrane potential (MMP) in KYSE410 and KYSE450 cells (Fig. 8f, g). Simultaneously, we observed an increasing Fe^2+^ concentration in KYSE410 and KYSE450 cells after adding D-lactate to these cells (Fig. 8h). Besides, we performed an intracellular glutathione (GSH) content measurement in KYSE410 and KYSE450 cells, which represents cellular redox state. We found that exogenous D-lactate was able to result in attenuated total GSH, reduced GSH and increased oxidized glutathione (GSSG) (Fig. 8i). These observations indicate that D-lactate may suppress stemness-related hallmarks through inducing esophageal CSCs death in an iron dependent pattern.

To figure out the role of CDK7-YAP-LDHD axis in the regulation of ferroptosis induced by D-lactate further, we added various concentrations of ferroptosis inducer Erastin to CDK7/YAP/LDHD-overexpressed and their control ESCC cells, then measured the sensitivity of these cells treated with Erastin for 24 h. According to the data from MTS analysis, we found that the up-regulation of CDK7-YAP-LDHD axis makes ESCC cells insensitive to Erastin treatment (Fig. 8j). Moreover, the overexpression of CDK7-YAP-LDHD axis led to decreased lipid ROS generation in ESCC cells (Supplementary Fig. 9d, e). Hence, increasing enrichment of CDK7-YAP-LDHD axis could attenuate the ferroptosis mediated by D-lactate in ESCC cells. In addition, when we added 1 μM ferroptosis inhibitor Fer-1 to CDK7, YAP or LDHD-depleted and their control KYSE410 cells, the spheroid-forming potential of these ESCC cells was restored (Supplementary Fig. 9f, g), suggesting that CDK7-YAP-LDHD axis could regulate ESCC cells’ stemness-associated properties by balancing D-lactate oxidation and ferroptosis occurrence.

## Discussion

Some studies have confirmed that the CSCs population exists in ESCC.^35,36^ However, the mechanisms of ESCC-CSCs stemness maintenance remain unknown. In this study, we revealed a novel machinery in which the nuclear CDK7-YAP complex was responsible for maintaining esophageal CSCs’ stem cell-like properties. Through screening multiple cell cycle-associated kinase suppressors, we observed CDK7 as a crucial regulator. CDK7 depletion led to decreased expression of stem cell-related molecules and attenuated potential for self-renewal, tumorigenicity, metastasis and chemotherapy resistance of ESCC-CSCs. We also demonstrated for the first time CDK7 could physically phosphorylate YAP at its Ser127 and Ser397 sites in the nucleus. As a consequence, such interaction between CDK7 and YAP promoted the transcription of YAP and its downstream target genes. It should be noted that these results were inconsistent with the previous research that YAP phosphorylation occurred at Ser127 and Ser397 depending on the canonical Hippo pathway upstream LATS1/2 kinases for cytoplasmic degradation.^37^ Interestingly, no matter whether CDK7 was overexpressed or reduced, Hippo-upstream genes LATS1, MST1, and the levels of LATS1/2 protein phosphorylation remained unchanged, indicating that the YAP phosphorylation mediated by CDK7 did not rely on Hippo pathway. However, the observation that nuclear phosphorylation of YAP-S127 and YAP-S397 enhanced YAP-induced transcriptional activity was consistent with the literature that Aurora A directly phosphorylated nuclear YAP at S397 and triggered YAP-induced transcription in triple-negative breast cancer cells.^38^ Hence, our data indicated that compartment-specific YAP phosphorylation at the same sites exerts different biological activities, suggesting an intricate YAP modulation.

Accumulating evidence demonstrates that CDKs possess broader roles.^9^ CDK7 not only participates in the phosphorylation of the canonical cell-cycle modulators involving in CDK1, CDK2, CDK4, and CDK6 but also activates noncanonical partners including P53^39^ and RNA polymerase II.^40^ During transcription initiation, CDK7 phosphorylates RNAPII C-terminal domain (RNAPII CTD) at Ser5 and Ser7, and CDK9 to activate transcription elongation.^41^ Furthermore, it was found that CDK7’s role in CSCs stemness maintenance mainly depended on its transcriptional regulatory function, which is distinct from its conventional function in cell cycle modulation. However, it should be noticed that CDK1, CDK4 and CDK6 were also the downstream substrates of CDK7, and they could modulate CSCs properties directly. Further research was necessary for determining if CDK7 was involved in maintaining CSC stemness via CDK4/6 or CDK1, in parallel with the CDK7-YAP axis.

Based on the previous vast literatures on the interaction between YAP transcriptional activity and multiple metabolic factors, we analyzed 2752 human metabolic enzymes and small molecule transporters through three RNA-seq data, then identified LDHD as a key targeted metabolic protein regulated by YAP in ESCC CSCs. In fact, most studies focused on LDHA among LDH family members, which mainly catalyzed the interconversion of pyruvate and L-lactate during glycolysis and gluconeogenesis pathway. Accumulated evidence has indicated that LDHA promotes differentiated tumor cells proliferation, invasion, metastasis, and drug-resistant via aerobic glycolysis in various solid tumors including prostate, osteosarcoma and gastric tumors.^42–44^ Here, for the first time, we identified the biological function of LDHD in ESCC cells. We observed that LDHD could promote tumor cell growth, migration, self-renewal, and tumorigenic potential in ESCC. Moreover, increasing LDHD protein status was associated with poor prognosis in clinical ESCC patients. LDHD serves as a moonlight oncoprotein that responds to the CDK7-YAP complex to form a pattern of the CDK7-YAP-LDHD axis in ESCC CSCs, ultimately modulating their stemness-related hallmarks.

Lactate was regarded as a kind of metabolic waste product derived from glycolysis previously until more and more functions of lactate were rediscovered in human tumors recent years. According to the “Lactate Shuttle” theory, L-lactate can shuttle between producer and consumer cells by acting as a main carbohydrate fuel functioning as a three-carbon compound or circulating redox buffer, a signaling molecule that participates in regulating cellular metabolism, immune response, and intercellular communication in tumor microenvironment niche, and a major gluconeogenic precursor.^45,46^ But for D-lactate, relevant researches in human tumors were minimal and its importance has always been ignored because of its low concentration at nanomoles level in serum physiologically.^47^ As a matter of fact, D-lactate content could soar to millimoles level in D-lactic acidosis, short bowel syndrome and tumors.^48,49^ This investigation revealed that D-lactate could repress tumor cell proliferation and self-renewal, while the equal L-lactate didn’t work. Therefore, we believed that esophageal CSCs were more sensitive to D-lactate compared with L-lactate despite its low concentration.

The term ferroptosis was coined by Dixon’s team in 2012 to describe an iron-dependent form of regulated cell death triggered by an overload of lipid peroxides on cellular membranes.^50^ Mechanically, GPX4 is identified as a central regulator of ferroptosis and its enzyme activity is blocked directly by aiming its selenocysteine site or the glutamate/cystine transporter system xCT indirectly, finally contributing to attenuated glutathione synthesis and lipid peroxidation, which induces the occurrence of ferroptosis.^51^ In this work, we found that excess D-lactate could repress xCT protein expression distinctly followed by decreased GPX4 and enhancive COX2 status. Moreover, accumulating D-lactate was able to induce tumor cells iron-dependent dead phenotypes involving in elevated ROS and intracellular iron content, while decreased MMP and GSH in ESCC. Hence, we were convinced that D-lactate elimination could protect esophageal CSCs from ferroptosis driven by CDK7-YAP-LDHD axis, ultimately supporting their stem cell-like properties.

In addition, we discovered that the pyruvate deriving from D-lactate catabolism could facilitate esophageal CSCs growth and self-renew. It’s well known that pyruvate is the principal carbon fuel input to support overall tricarboxylate cycle carbon flux and is produced from different sources such as the oxidation of mitochondrial lactate, the end product of glycolysis, and the transamination of alanine.^52^ Endogenous pyruvate fuels mitochondrial respiration to heighten breast cancer cells proliferation and these cells also depend on environmental pyruvate to shape the metastatic niche.^53^ Besides, pyruvate released from patient-derived cancer-related fibroblasts promotes the survival of primary lymphoma cells.^54^ Interestingly, there were some controversial opinions about exogenous pyruvate and some argued that exogenous pyruvate suppressed tumor cells proliferation by downregulating the expression of histone-associated genes,^55^ while others confirmed that exogenous pyruvate facilitated cancer cells adaptation to hypoxic niche for enhancing proliferative potential and they thought that some metabolic intermediates in citric acid cycle perhaps were more essential than pyruvate derived from glycolysis.^56^ Consistently, our study also revealed that some metabolites such as succinate and alanine were increased distinctly following the elevated pyruvate originated from D-lactate mediated by CDK7-YAP-LDHD axis in mitochondria. Furthermore, our work also testified that exogenous pyruvate was capable to support stemness-related properties in ESCC CSCs.

Cumulatively, our findings revealed a new mechanism that the specific phosphorylation of nuclear YAP at its S127 and S397 sites mediated by CDK7 promotes LDHD transcription to drive D-lactate catabolism in the mitochondria, protecting esophageal CSCs from ferroptosis and generating pyruvate, finally supporting stem cell-like hallmarks of ESCC CSCs (Fig. 8k). Therefore, CDK7-YAP-LDHD axis might be an attractive candidate for ESCC therapy by targeting metabolic checkpoint.

## Materials and methods

### Cell culture and tissue specimens

Dr. Y Shimada (Kyoto University) gifted human ESCC cell lineage, which were cultured in RPMI 1640 culture medium (Lonza, Switzerland) containing 1% penicillin/streptomycin and 10% fetal bovine serum (Gibco, Grand Island, NY, USA) in an incubator (37 °C, 5% CO_2_). The ESCC specimens were acquired from Shanghai Outdo Biotech Co. Ltd., and the patients were first informed and then their consent was taken. The investigation was authorized by the Research Ethics Board of Peking University Cancer Hospital.

### Plasmid construction and lentivirus package

The whole coding sequence of *YAP*, *CDK7*, and *LDHD* was cloned into lentiviral vector (pLenti6 or GV492) using DNA recombinant method. The construct and package (pMD2.G, psPAX2) plasmids were transfected into HEK293T cells using Lipofectamine 2000 (Invitrogen, Carlsbad, CA) for lentivirus package. ESCC cells involving in KYSE140, KYSE150, KYSE410, and KYSE450 were transfected. For non-targeting control, an empty vector construct was utilized. For *YAP*, *CDK7*, and *LDHD* knockdown, their specific short hairpin RNA (shRNA) sequences were subcloned into pCDH or GV493 vector, as well as corresponding scramble shRNA sequences, which were used as shRNA non-targeting control groups. Stable clones were identified with 2 μg/ml puromycin (Sigma-Aldrich, St Louis, MO, USA). Supplementary Table 1 enlist all the shRNA information. Cells were transfected transiently using their specific siRNA with the help of lipofectamine 2000 (Invitrogen). Supplementary Table 2 enlist all the siRNA information.

### Sphere formation assay

ESCC cells were seeded into ultra-low attachment ninety-six or six-well culture plates (Corning Incorporated Life Sciences, Acton, MA, USA) and propagated in DMEM/F12 media (Gibco) containing 1% methylcellulose (Sigma-Aldrich), 1 × B27 (Invitrogen), and 20 ng/ml of epidermal growth factor and basic fibroblast growth factor (Gibco). Cells were propagated for 2 weeks, and the representative micrographs of these spheres were acquired using the stereomicroscope and their diameter was assessed via the LAS V4.9 software (Leica, Germany).

### Animal assays

Almost 2 × 10^7^ KYSE410 cells were administered into BALB/C nude mice (4–6 weeks old, females). After one week, the animals were randomly categorized into four groups treated as: (1) 0.9% NaCl, (2) cisplatin (5 mg/kg, administered intraperitoneally per week), (3) THZ1 (10 mg/kg, administered intraperitoneally daily, Selleck, S7549), (4) cisplatin + THZ1 (4 weeks). For 4 weeks, tumor size was assessed every 4 days. For tumorigenicity assay in vivo, 1000 and 100 cells were suspended in 50 μl RPMI 1640 medium with equal amount of Matrigel (BD, Biosences, Bedford, MA, USA), and were subcutaneously transplanted into the back of non-obese diabetic/severe combined immunodeficient (NOD/SCID) mice (4–6 weeks old, females) (HFK BIOSCIENCE Co. Ltd., Beijing, China). Around 4–6 weeks after injection, the tumors were dissected and weighed. The animal protocols were authorized by the Institutional Research Animal Care and Use Committee of Peking University Cancer Hospital.

### RNA extraction and quantitative real-time PCR

Whole RNA was separated via TRIZOL Reagent (Invitrogen, 15596026), and utilized for generating cDNA with the help of RevertAid First Strand cDNA Synthesis Kit (ThermoFisher Scientific, K1691). SYBR® Premix Ex Taq™ (Tli RNaseH Plus) Kit (Takara, RR420A) was utilized for performing real time qPCR on an ABI PRISM 7500 Sequence Detector (Applied Biosystems). mRNA expression alterations were measured by the 2^−△△CT^ formula. GAPDH was set as endogenous control. Supplementary Table 3 enlists primers detail.

### Western blot analysis

Cell lysis was carried out using RIPA buffer (Beyotime, China, P0013E), supplemented with phosphatase inhibitor and protease inhibitor cocktail (Roche, Germany). The quantified protein was separated using sodium dodecyl sulfate-polyacrylamide gel electrophoresis and transferred into polyvinylidene fluoride membranes (Millipore, Billerica, CA, USA). The primary antibodies used in this study are listed in Supplementary Table 4. Protein bands were detected using the Amersham Imager 600 (GE, America).

### Immunofluorescence

Cells were propagated on a glass-bottomed culturing dish (Nest, China) for 24 h, preserved using 4% paraformaldehyde, permeabilized for 10 min by 0.04% Triton X-100, blocked in 10% normal goat serum (ZSGB-BIO, China), labeled at 4 °C with primary antibodies overnight, washed, tagged for 1 h with secondary antibodies, and stained by DAPI for 15 min at ambient temperature (1:10000, Beyotime, C1002). Photographs were acquired using a confocal microscope (Leica ST2, Germany). Supplementary Table 4 indicates the primary antibodies utilized.

### Immunohistochemistry

The tissue specimens were deparaffinized, hydrated, and their antigen was retrieved by boiling (3 min in electric pressure cooker with Tris-EDTA buffer, pH 9.0). Then the sections were blocked using 10% normal goat serum (ZSGB-BIO), labeled at 4 °C with anti-LDHD antibody (1:100, Proteintech, 14398-1-AP) overnight, tagged with a secondary antibody and then stained with DAB (Dako, Denmark). The staining intensity was scored using the following scale: 0 for negative, 1 for weak, 2 for moderate, 3 for strong, and 4 for very strong.

### Chromatin immunoprecipitation

Chromatin immunoprecipitation was performed with Pierce Magnetic ChIP Kit (ThermoFisher, 26157) according to the manufacturer’s instructions. Briefly, KYSE410 cells were fixed with 1% formaldehyde for 10 min, followed by glycine quenching, lysis and MNase digestion. Immunoprecipitation was performed with 10 μg anti-YAP (Cell Signaling Technology, 14074 S), anti-TEAD1 (Abcam, ab133533), anti-TEAD2 (Proteintech, 21159-1-AP) or anti-TEAD4 (Abcam, ab197589) antibodies overnight at 4 °C, followed by washing, elution and reverse crosslinking. The immunoprecipitated DNA was quantified by qRT-PCR.

### Co-immunoprecipitation

Cells were collected by scraping and lysed with 1% NP-40 lysis buffer (Beyotime, P0013F) containing 50 mM Tris-HCl (pH = 7.4), 150 mM NaCl, 5 mM EDTA (pH = 7.4) and complete protease inhibitor cocktail and phosphatase inhibitor (Roche) at 4 °C for 30 min. Lysates were discarded using centrifugation. 100 μl supernatant was used as an input and the left were incubated with protein A/G- magnetic beads (NCM Biotech, China) overnight at 4 °C. 5 μg anti-CDK7 (Abcam, ab243863), anti-YAP (Cell Signaling Technology, 14074S) or anti-LDHD (Proteintech, 14398-1-AP) antibody and rabbit or mouse IgG were added to the supernatant and incubated at 4 °C for 4-6 h. The complex was washed for three times with 1% NP-40 lysis buffer and the beads were boiled with loading buffer for 10 min.

### Proximity ligation assay

The PLA was performed using in situ Proximity Ligation Assay kit (Sigma-Aldrich, DUO92008) according to the manufacturer’s instructions. In brief, about 1–4 × 10^5^ KYSE410 cells were seeded in a confocal dish. Anti-YAP antibody (30 μg/ml, Sigma-Aldrich, WH0010413M1) and anti-CDK7 antibody (1:50, Abcam, ab243863) were added to the cells and incubated overnight. The nuclei were stained with DAPI (1:10,000, Beyotime, C1002) for 15 min and micrographs were obtained using a confocal microscope (Leica ST2).

### Kinase assay in vitro

For kinase assay in vitro, 12 μg CDK7-Flag and YAP-Flag plasmids were transfected into HEK293T cells and incubated for 60 h, respectively. Collected these cells and added 1 ml 1% NP-40 lysis buffer (Beyotime) containing protease and phosphatase inhibitor (Roche). Then obtained the supernatant and added 30 μl Protein A/G beads (NCM Biotech) to incubate at 4 °C overnight. The next day, added 5 μg anti-Flag antibody to each reaction and incubated at 4 °C for 4 h. Washed the beads with 500 μl 1 × kinase reaction buffer (25 mM Tris pH 7.5, 2 mM DTT, 10 mM MgCl_2_, 200 μM ATP) containing protease and phosphatase inhibitor (Roche) for three times. Then added 10 μg YAP-beads and 0.5 μg CDK7-beads to 100 μl reaction system and incubated at 30 °C water bath for 30–60 min. Boiled water bath for 10 min was used to terminate the reaction and then froze with liquid nitrogen. Subsequent mass spectrometric identification and data analysis were completed by SpecAlly Life (Wuhan) Science and Technology Co. Ltd.

### 2-NBDG up-take assay

Intercellular glucose was measured with the fluorescently-labeled glucose analog 2-[N-(7-nitrobenz-2-oxa-1,3-diazol-4-yl) amino]-2-deoxy-D-glucose (2-NBDG, Invitrogen, N13195). Approximately 1 × 10^6^ cells were seeded in a six-well culture plate and then starved by adding glucose-free RPMI-1640 containing 10% FBS. At 16–24 h later, the culture medium was discarded and 100 μM 2-NBDG was added, then the cells were incubated at 37 °C in a 5% CO_2_ humidified incubator for 30 min. The cells were collected and the fluorescence intensity was analyzed using BD Accuri™ C6 flow cytometer (BD Biosciences) after the labeled cells were filtered through a 40-μm nylon mesh.

### Untargeted metabolomic mass spectrometry analysis

For metabolites measurement, 5 × 10^6^ control KYSE410 cells infected with empty lentivirus and YAP or LDHD overexpressing KYSE410 cells were seeded in 10 cm cell culture dishes, respectively. After 24 h, cells were washed with cold 1 × PBS three times and digested into single-cell suspension. Counted 0.5–1 × 10^7^ cells for each group and transferred them into a 1.5 ml tube, then froze quickly with liquid nitrogen and stored at −80 °C. For isotope labeling experiments, 5 × 10^6^ wild type KYSE410 cells were incubated with RPMI-1640 medium without glycine for 12 h and changed to medium containing 400 μM [U-^13^C_2_] glycine or complete culture medium, which served as a blank control group. Cells were washed rapidly and lysed 12 h later, then froze under liquid nitrogen as fast as possible. Mass spectrometry data analysis was performed by the Biotree Biotech (Shanghai, China).

### D-lactate and pyruvate assay

Basal D-lactate production was measured using D-Lactate Assay Kit (Biovision, K667) according to the manufacturer’s instructions and Pyruvate Assay Kit (Sigma-Aldrich, MAK071) was used for pyruvate assay.

### ROS generation assay

BODIPY 581/591 C11 (MedChemExpress, HY-D1301), as a lipid peroxidation sensor, was used to detect ferroptosis. 1 × 10^5^ KYSE410 and KYSE450 cells were seeded in six-well culture plate supplementing 8 mM and 16 mM D-lactate and incubated at 37 °C for 72 h. Then 5 μM BODIPY 581/591 was added to cells and incubated at 37 °C for 30 min. Washed cells with 1 × PBS for three times and collected cells for flow cytometry analysis with FL1 channel.

### Mitochondrial membrane potential assay

Lipophilic fluorescent dye JC-1 (MedChemEXpress, HY-K0601) was used to measure the changes of mitochondrial membrane potential. Briefly, 1 × 10^5^ KYSE410 and KYSE450 cells were resuspended and seeded in six-well culture plate with 1 ml RPMI-1640 culture media containing 8 mM and 16 mM D-lactate. 72 h later, added 10 μl JC-1 to cells for the concentration at 2 μM and incubated at 37 °C for 20 min. CCCP was used as a positive control and added 1 μl CCCP to cells for a final concentration at 50 μM. Then incubated cells at 37 °C for 5 min. Washed cells with 1 × PBS for three times and collected cells to analyze on a flow cytometer (BD Biosciences, Accuri^TM^ C6).

### Fe^2+^ concentration assay

For intracellular Fe^2+^ content measurement, about 5 × 10^6^ cells were seeded in 10 cm dishes supplementing 8 mM and 16 mM D-lactate for KYSE410 and KYSE450 cells, respectively. After 72 h, collected cells and tested Fe^2+^ content using Intracellular Iron Colorimetric Assay Kit (Applygen, E1042) according to the manufacturer’s instructions.

### GSH and GSSG assay

About 5 × 10^6^ cells were seeded in 10 cm dishes supplementing 8 mM and 16 mM D-lactate for KYSE410 and KYSE450 cells to measure intracellular glutathione status. 72 h later, collected these cells and detected total GSH, reduced GSH and GSSG content in these cells by a GSH and GSSG Assay Kit (Beyotime, S0053) according to the manufacturer’s instructions.

### Statistics

Statistical measurements were carried out via SPSS Statistical 17.0. Intergroup comparison was performed by the Two-tail Student’s *t* test. Kaplan Meier survival test was carried out to elucidate the survival association of LDHD expressions and the significance of the differences was assessed by the log-rank test. Tumorigenic cell frequency was calculated based on extreme limiting dilution analysis using the webtool at http://bioinf.wehi.edu.au/software/elda/ (Hu and Smyth, 2009). *p* ≤ 0.05 was considered statistically significant.

### Supplementary information

Supplementary Information

## Data Availability

The raw sequence data reported in this paper have been deposited in the Genome Sequence Archive (Genomics, Proteomics & Bioinformatics 2021) in National Genomics Data Center (Nucleic Acids Res 2022), China National Center for Bioinformation/Beijing Institute of Genomics, Chinese Academy of Sciences (GSA-Human: HRA004317) that are publicly accessible at https://ngdc.cncb.ac.cn/gsa-human. Further information and requests for resources and reagents should be directed to and will be fulfilled by the corresponding author, Q.Z. (zhanqimin@bjmu.edu.cn).
