# Supplementary material for: CDK7-YAP-LDHD axis promotes D-lactate elimination and ferroptosis defense to support cancer stem cell-like properties
Source: Signal Transduct Target Ther. 2023 Aug 16;8:302. doi: 10.1038/s41392-023-01555-9 (PMC10427695; doi:10.1038/s41392-023-01555-9)
Supplement: Supplementary file 1 — Supplementary Information [file 41392_2023_1555_MOESM1_ESM.docx]

Supplementary Materials for

CDK7-YAP-LDHD axis promotes D-lactate elimination and ferroptosis defence to support cancer stem cell-like properties

Mengzhu Lv^1,#^, Ying Gong^1,2,#^, Xuesong Liu^1,#^, Yan Wang^1,4^, Qingnan Wu^1,4^, Jie Chen^1,4^, Qingjie Min^1^, Dongyu Zhao^5^, Xianfeng Li^1^, Dongshao Chen^1^, Di Yang^1^, Danna Yeerken^1^, Rui Liu^1^, Jinting Li^1^, Weimin Zhang^1,3,4,7,*^, Qimin Zhan^1,3,4,5,6,*^

Correspondence to: zhanqimin@bjmu.edu.cn, zhangweimin@bjmu.edu.cn

**This PDF file includes:**

Materials and Methods

Figures. S1 to S9

Tables S1 to S4

Materials and Methods

**RNA-seq**

For RNA-seq analysis, about 1×10^7^ ESCC cells were used for total RNA extraction for each sample. Washed the cells with cold 1×PBS for three times and scraped the cells gently. Transferred the cell pellets into a new tube and added 1 ml TRIZOL Reagent (Invitrogen, 15596026) for each sample, then froze at -80°C. RNA library establishment, sequencing and data analysis were performed by Novogene Bioinformatics Technology Co. Ltd. High-throughput sequencing was carried out on a Hiseq X Ten platform to produce 150 bp paired-end reads.

**Flow cytometry analysis**

Added 20 μM cisplatin to ESCC cells and incubated them for 48 h. These cells were then stained with propidium iodide (PI) and Annexin-V-APC acquired from the Annexin V/PI apoptosis detection kit (Dojindo, Japan, AD10) following the manufacturer’s guidelines. The ratio of apoptotic cells was analyzed by Accuri™ C6 flow cytometer (BD Bioscience).

**Cell cycle assay**

For cell cycle analysis, cells were fixed overnight at 4°C with 70% ethanol. Then these cells were stained for 15 min with PI (BD Biosciences, 550825) at 25°C, followed by analyzing and assessing their cell cycle distribution or DNA content using Accuri™ C6 flow cytometer (BD Bioscience).

**Colony formation assay**

Counted and resuspended about 5×10^3^ cells with complete medium, then propagated these cells into 60 mm plates and cultured them for 2 weeks. The grown colonies were subsequently fixed with 100% methanol for 10 min. 0.1% crystal violet solution was used to stain the colonies for 30 min. Photographs of these colonies were acquired by a stereomicroscope (Leica).

**Scratch wound healing assay**

LDHD knocked-down or overexpressed KYSE150 and KYSE450 cells (5×10^5^) and the control ESCC cells were propagated in 6-well plate for 24 h. After adherence, the cells were scratched using a scratcher crosswise and formed wounds. Cells proliferation status around the scratch wound was observed and over three fields were randomly selected for imaging and statistics at 24 h, 48 h and 72 h, respectively.

**Migration assay**

Cells were resuspended with FBS-free medium and then planted into the top chamber of a 24-well insert (8μm, Corning), while the bottom chamber was filled with medium containing 20% FBS as an attractant. Incubated these cells for 24 h and fixed them with 100% methanol, followed by staining for 30 min with 0.1% crystal violet. The migrated cells were visualized and analyzed using a stereomicroscope (Leica).

**Mitochondria isolation and protein extraction**

For mitochondrial protein extraction, we first isolated the mitochondrion of ESCC cells using Cell Mitochondria Isolation Kit (Beyotime, C3601) according to the manufacturer’s instructions. Approximate 2×10^7^ KYSE410 and KYSE450 cells were used in the assay. Added 1 ml mitochondrial separation reagent with 1 mM phenylmethylsulfonyl fluoride (PMSF) to cells and incubated on ice for 15 min. Treated the cell suspension by homogenate for about 15 times. After a series of density gradient centrifugation, added 100 μl mitochondrial lysis buffer containing 1 mM PMSF to cells and incubated on ice for 30 min. The supernatant was used for Western blot analysis.

**
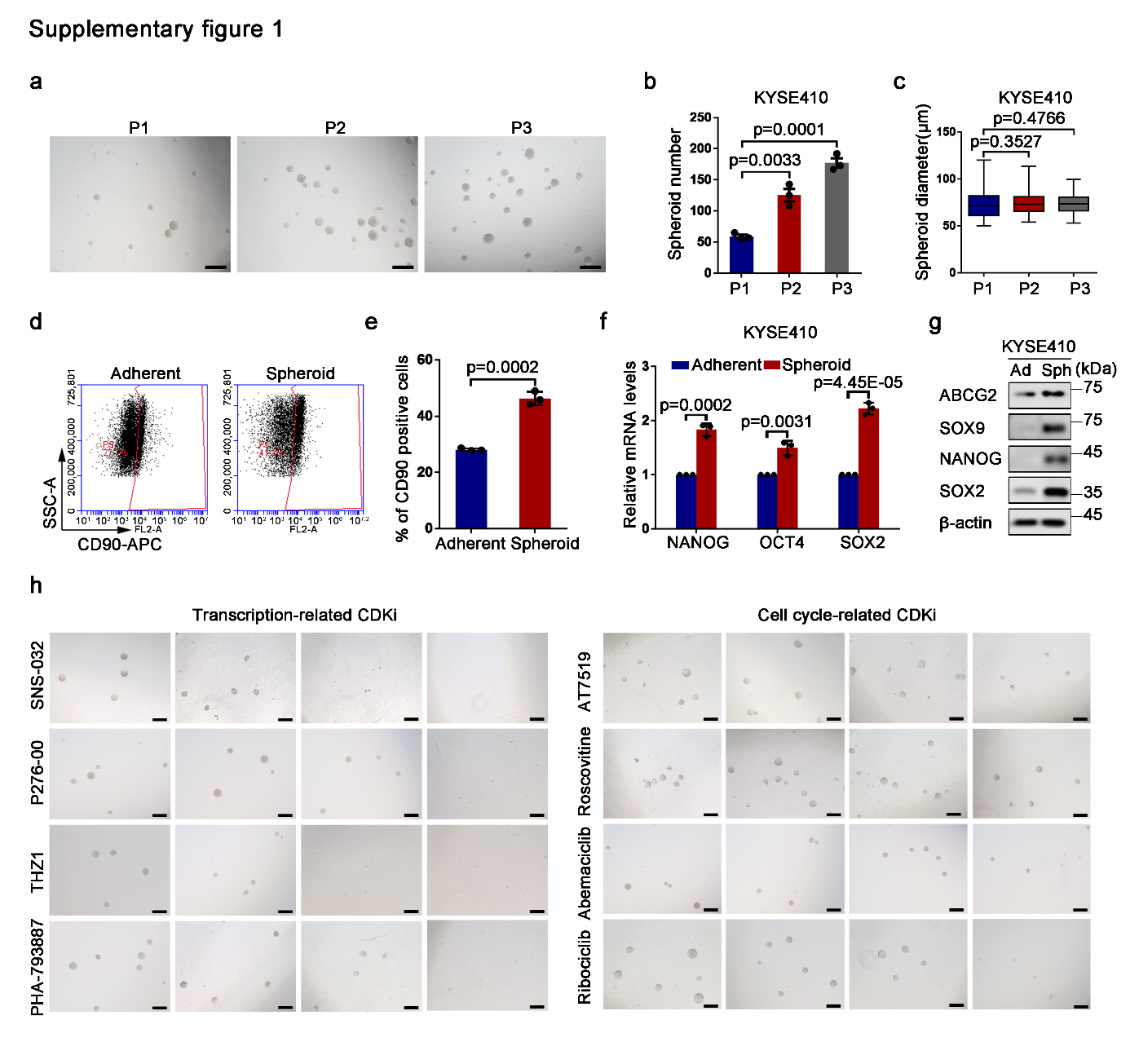
**

**Figure. S1. A small-molecule screen identified CDK7 inhibitor THZ1 with powerful anti-CSCs properties.**

**a** Representative picture of spheres from serial passages (P1, P2 and P3) of KYSE410 cells. Scale bar, 200 μm. **b** The number of spheres per well in (**a**). **c** The diameter of spheres per well in (**a**). **d** The percentage of CD90 high/low populations was analyzed by flow cytometry in adherent KYSE410 cells and KYSE410 spheres and (**e**) percentage of CD90 high/low populations was quantified and graphed. **f** Total RNA of adherent KYSE410 cells and KYSE410 spheres was extracted and the expression of stemness-associated genes (*NANOG, OCT4, SOX2*) was measured by qRT-PCR. **g** Western blot analysis for the expression of stemness-indicated molecules in adherent (Ad) KYSE410 cells and KYSE410 spheres (Sph). β-actin was used as an internal reference. **h** Sphere formation of KYSE410 cells treated with small molecule inhibitors with the indicated concentrations for 14 days. KYSE410 cells were treated with transcription-related CDKi (left) and cell cycle-related CDKi (right). Scale bar, 200 μm. Error bars represent mean ± SD (n=3) in (**b**), (**c**), (**e**) and (**f**).


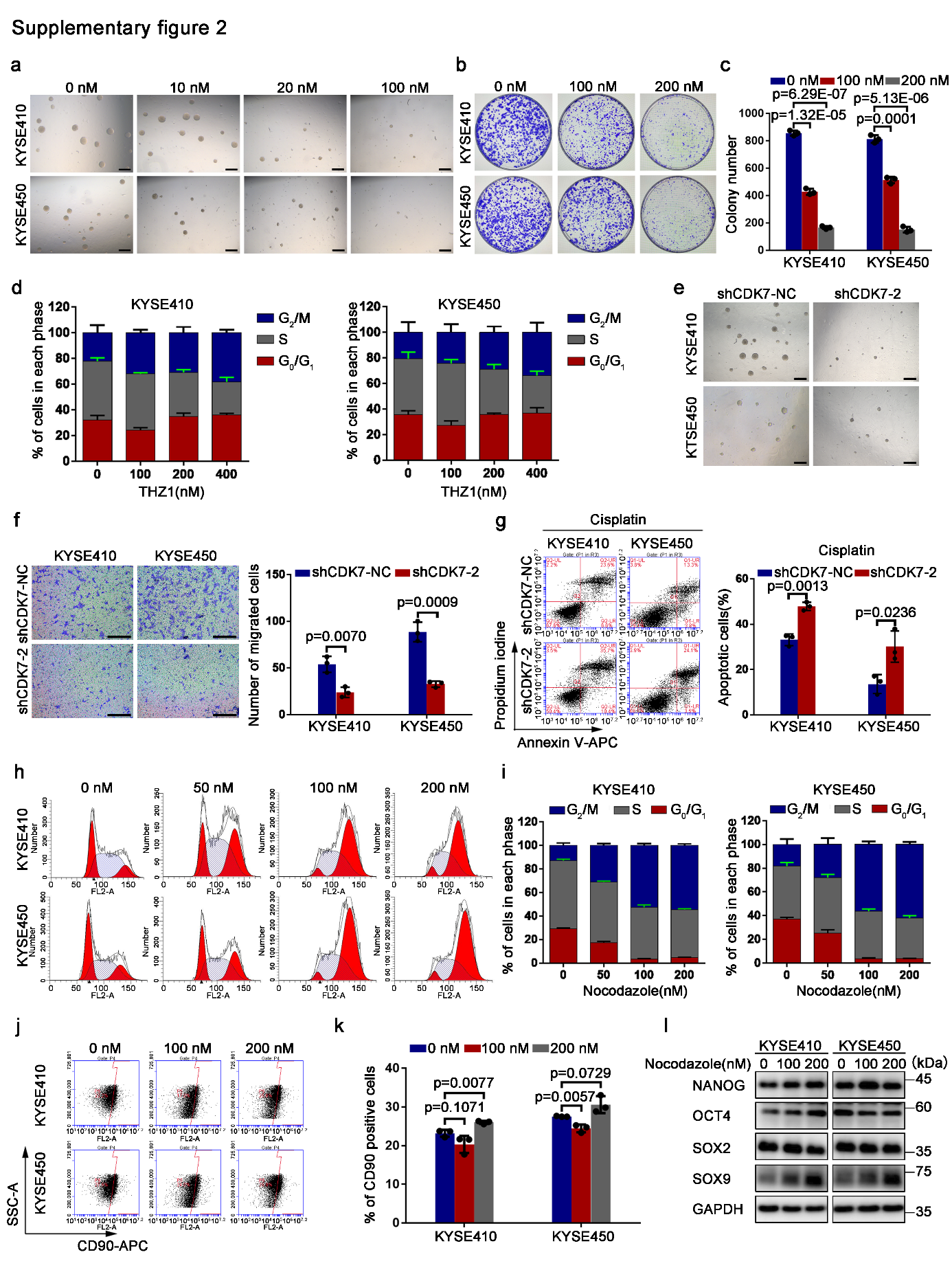


Figure. S2. CDK7 modulates ESCC cells stemness-associated properties independently of its role in the G_2_/M transition.

**a** Representative image of spheres of KYSE410 and KYSE450 cells cultured in the RPMI-1640 medium containing THZ1 at indicated concentrations. Scale bar, 200 μm. **b** Results of colony formation assays for KYSE410 and KYSE450 cells treated with THZ1 for 14 days. **c** The cell colonies in (**b**) were quantified and graphed. **d** KYSE410 and KYSE450 cells were treated with the indicated concentrations of THZ1 for 48 h and analyzed by ﬂow cytometry after staining with propidium iodide (PI). These cells at different cell cycle phases were quantified and graphed. **e** Representative images of spheres from shCDK7-transfected (shCDK7-2) and control (shCDK7-NC) KYSE410 and KYSE450 cells. Scale bar, 200 μm. **f** Representative images of migrated shCDK7-transfected (shCDK7-2) and control (shCDK7-NC) KYSE410 and KYSE450 cells (left). Scale bar, 200 μm. These cells were quantified and graphed (right). **g** shCDK7-transfected (shCDK7-2) and control (shCDK7-NC) KYSE410 and KYSE450 cells were treated with cisplatin for 48 h. Apoptotic cells were stained by annexin V and PI (left) and the apoptotic KYSE410 and KYSE450 cells were quantified and graphed (right). **h** KYSE410 and KYSE450 cells were treated with the indicated concentrations of Nocodazole for 48 h and analyzed by ﬂow cytometry after staining with propidium iodide (PI). **i** KYSE410 (left) and KYSE450 (right) cells at different cell cycle phases were quantified and graphed. **j** KYSE410 and KYSE450 cells treated with the indicated concentrations of Nocodazole for 48 h were labeled with CD90-APC antibody and CD90 high/low populations were analyzed by flow cytometry. **k** The percentage of CD90 high/low populations was quantified and graphed. **l** Immunoblotting analysis of NANOG, OCT4, SOX2 and SOX9 protein levels in KYSE410 and KYSE450 cells which were treated with Nocodazole at the indicated concentrations. GAPDH was used as an internal reference. Data were presented as mean ± S.D (n=3).


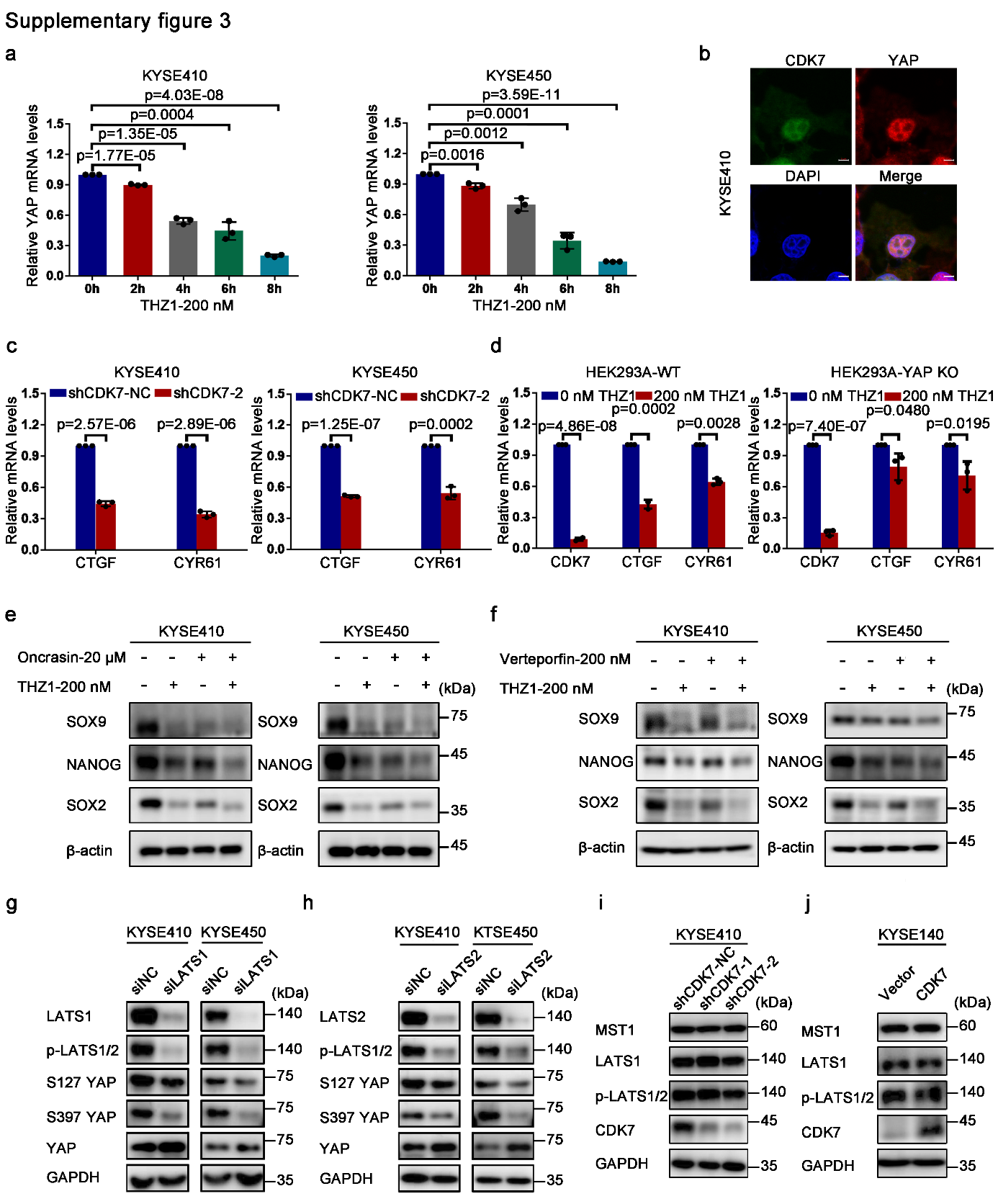


Figure. S3. CDK7-YAP complex regulates esophageal CSCs properties independently of Hippo pathway.

**a** Real-time PCR analysis of YAP mRNA expression in KYSE410 (left) and KYSE450 (right) cells cultured in the RPMI-1640 medium containing THZ1 for indicated time. **b** Immunofluorescent images representing the nuclear/cytoplasmic localization of CDK7 (Alexa Fluor 488, green) and YAP (Alexa Fluor 543, red) in KYSE410 cells, DAPI (blue) was used for the nuclear stain. Scale bar, 5 μm. **c** Real-time PCR analysis of YAP target genes in shCDK7-transfected (shCDK7-2) and control (shCDK7-NC) KYSE410 and KYSE450 cells. **d** Real-time PCR analysis of YAP target genes in YAP-depleted and wild type HEK293A cells treated with 200 nM THZ1 for 36 h. **e** Western blot analysis for the expression of indicated molecules in RNAPII-depleted (20 μM Oncrasin, 24 h) and wild type ESCC cells treated with 200 nM THZ1 for 36 h. **f** Immunoblotting analysis of stemness-associated indicators in YAP-depleted (200 nM Verteporfin, 72 h) and wild type KYSE410 and KYSE450 cells treated with 200 nM THZ1 for 36 h. **g** The expression of related protein when LATS1 was knocked down by siRNA in KYSE410 and KYSE450 cells. **h** The expression of related proteins when LATS2 was knocked down by siRNA in KYSE410 and KYSE450 cells. **i** The expression of related proteins in shCDK7-transfected (shCDK7-1, shCDK7-2) and control (shCDK7-NC) KYSE410. **j** The expression of related proteins in CDK7 and empty vector transfected KYSE140 cells. GAPDH or β-actin was used as an internal reference in immunoblotting analysis. Error bars in (**a**), (**c**) and (**d**) represent the mean ± S.D. of three independent experiments (n=3).


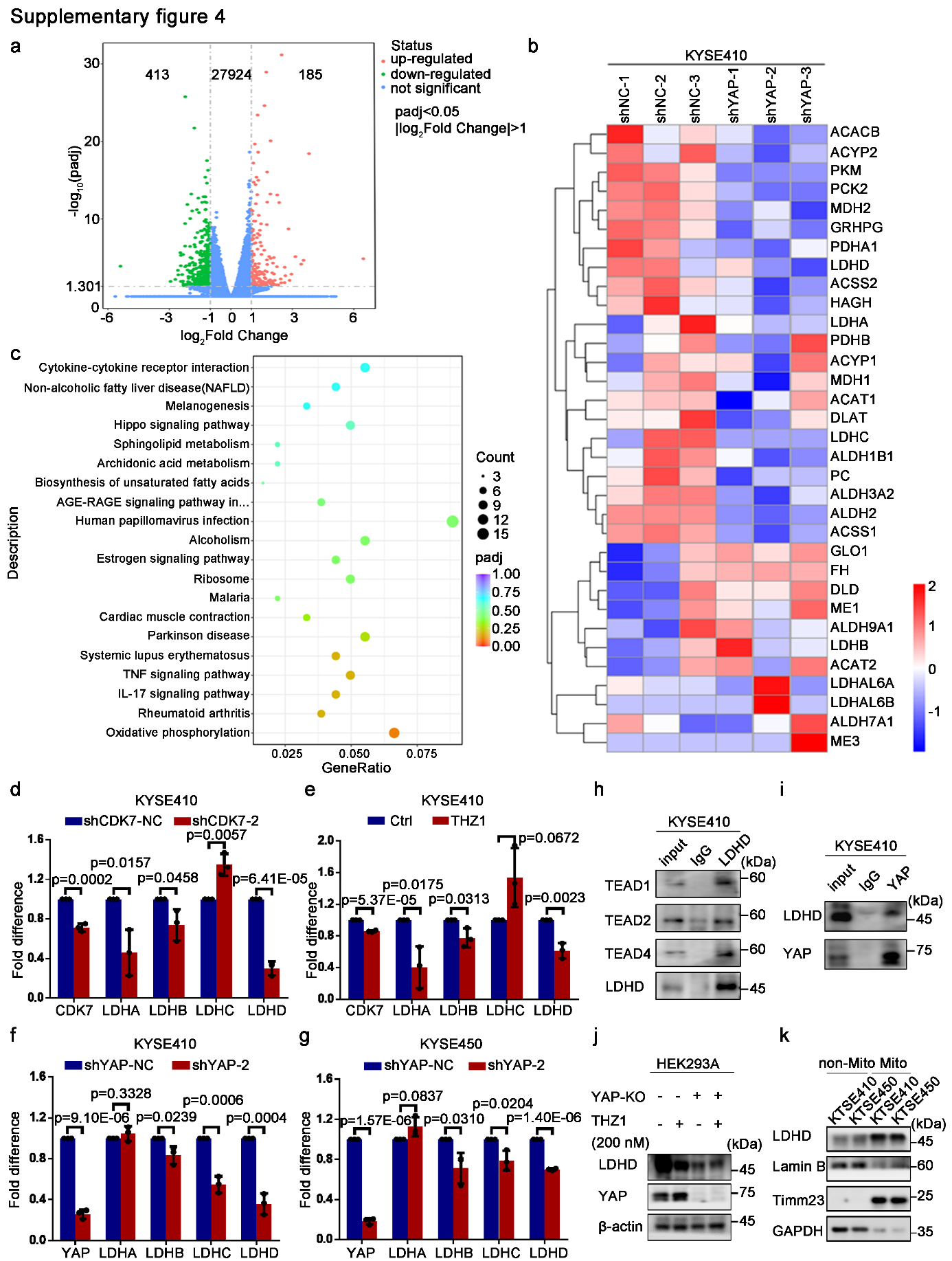


Figure. S4. CDK7-YAP axis positively regulates the expression of LDHD in ESCC CSCs.

**a** Volcano plot showing differentially expressed genes in KYSE410 cells following knocking down YAP. **b** Heatmap displaying differentially expressed genes associated with pyruvate metabolism in KYSE410 cells after interfering YAP. **c** Dot plot suggesting alternative signaling pathways when the expression of YAP was suppressed in KYSE410 cells. **d** Real-Time PCR analysis for the expression of LDH family in KYSE410 cells with attenuated CDK7 status. **e** Real-Time PCR measurement for the expression of LDH members in KYSE410 cells treated with 200 nM THZ1 for 4 h. **f-g** Histogram indicating the mRNA expression of LDH family in KYSE410 and KYSE450 cells after knocking down YAP with specific shRNAs. **h** Co-immunoprecipitation analysis showing the interaction of LDHD and TEAD protein in KYSE410 cells. **i** Co-immunoprecipitation analysis for the interaction between YAP and LDHD in KYSE410 cells. **j** Western blotting analysis showing the expression of LDHD in YAP-depleted and control HEK293A cells treated with 200 nM THZ1 for 36 h. β-actin was used as an internal reference. **k** Western blot result suggesting the expression of LDHD protein in non-mitochondrial and mitochondrial component. Lamin B, Timm23 and GAPDH were used as the internal reference of nucleus, mitochondria and cytoplasm. Error bars represent mean ± SD (n=3).


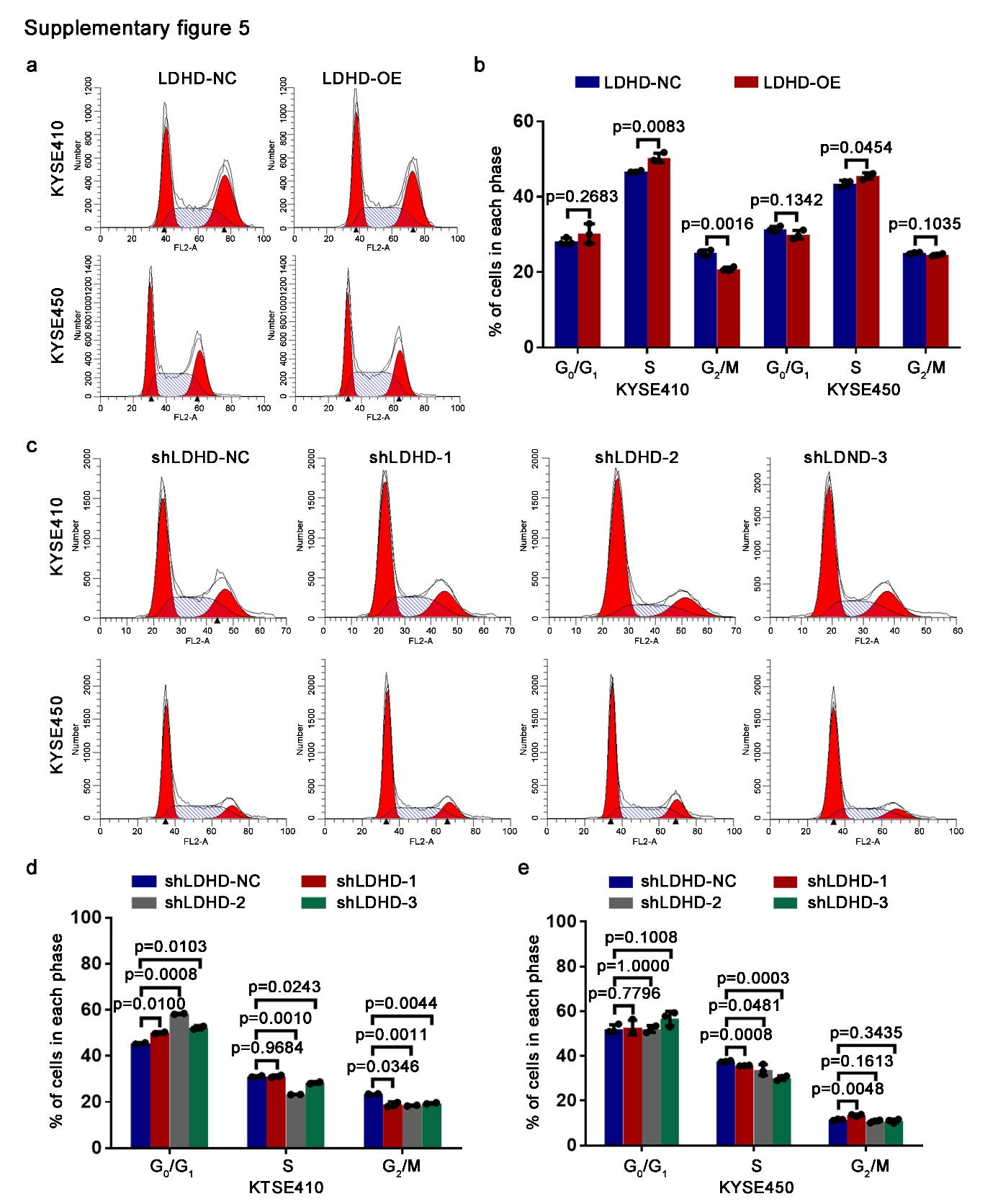


Figure. S5. LDHD is responsible for tumor cells proliferation in ESCC.

**a** Cell cycle analysis for LDHD-overexpressed KYSE410 and KYSE450 cells labeled using PI with a flow cytometry illustrating the ratio of cellular distribution in each phase and (**b**) data were analyzed and graphed as a histogram. **c** Diagram of cell cycle distribution showing the effect of decreased LDHD expression on the ratio of cells in each phase. **d-e** Histogram indicating the changes for cell cycle distribution mediated by reduced LDHD expression in KYSE410 and KYSE450 cells. Data in (**b**), (**d**) and (**e**) were presented as mean ± S.D (n=3).


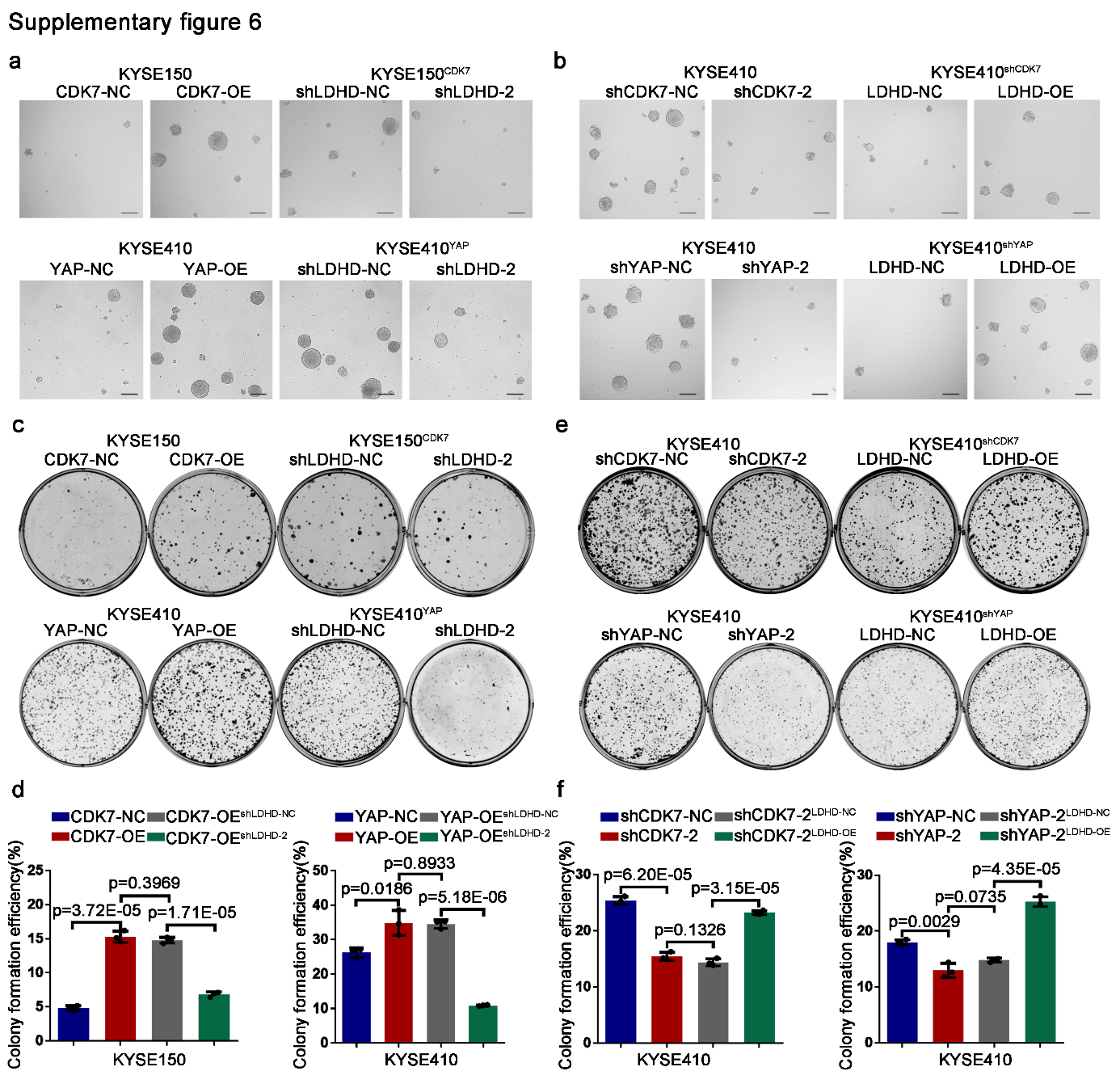


Figure. S6. CDK7-YAP-LDHD axis accelerates tumor cells growth in ESCC.

**a** Representative photograph of spheroids formed by CDK7-overexpressed KYSE150 (top) and YAP-overexpressed KYSE410 (bottom) cells after knocking down the expression of LDHD by their shRNAs. Scale bar, 100 μm. **b** Representative graphs displaying spheres formed by CDK7-knocked down (top) or YAP-knocked down (bottom) KYSE410 cells following forced LDHD expression by transfected with their lentivirus. Scale bar, 100 μm. **c** Representative images of colonies formed by CDK7-overexpressed KYSE150 (top) and YAP-overexpressed KYSE410 (bottom) cells with decreased LDHD expression and (**d**) these colonies were counted and graphed as histograms. **e** Photographs showing the colonies formation capacity of CDK7-knocked down (top) and YAP-knocked down (bottom) KYSE410 cells after overexpressing LDHD. **f** Histogram demonstrating the colony formation efficiencies of these cells transfected with lentivirus containing LDHD cassette. Error bars in (**d**) and (**f**) represent mean ± SD (n=3).


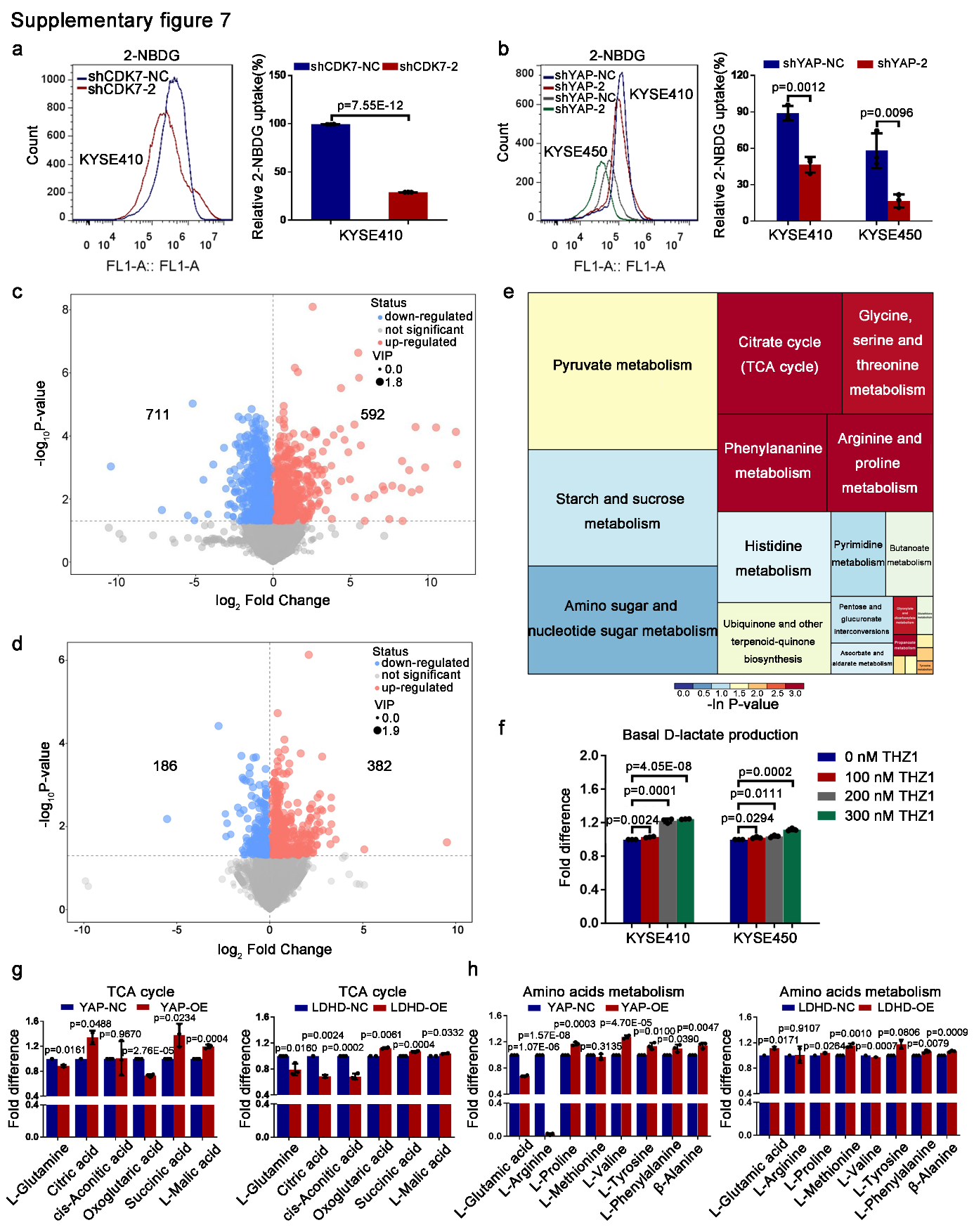


Figure. S7. CDK7-YAP-LDHD axis drives metabolic reprogramming in esophageal CSCs.

**a** Flow cytometry analysis for 2-NBDG uptake in CDK7-knocked down KYSE410 cells (left) and histogram showing relative glucose uptake ratio following decreased CDK7 (right). **b** Flow cytometry assay for alternative 2-NBDG uptake in YAP-knocked down KYSE410 and KYSE450 cells (left). Relative glucose uptake ratio was calculated and graphed as the histogram (right). **c** Volcano plot showing differentially existed metabolites in YAP-overexpressed and control KYSE410 cells. **d** Volcano plot hinting changed metabolites after overexpressing LDHD in KYSE410 cells. **e** Tree plot showing several altered metabolic pathways after overexpressing LDHD in KYSE410 cells. **f** Histogram demonstrating changed D-lactate content in KYSE410 and KYSE450 cells treated with indicated concentrations of THZ1 for 4 h. **g-h** Untargeted metabolomic mass spectrometry analysis for alternative metabolites in TCA cycle and amino acids metabolism after overexpressing YAP or LDHD in KYSE410 cells. Error bars represent mean ± SD (n=3).


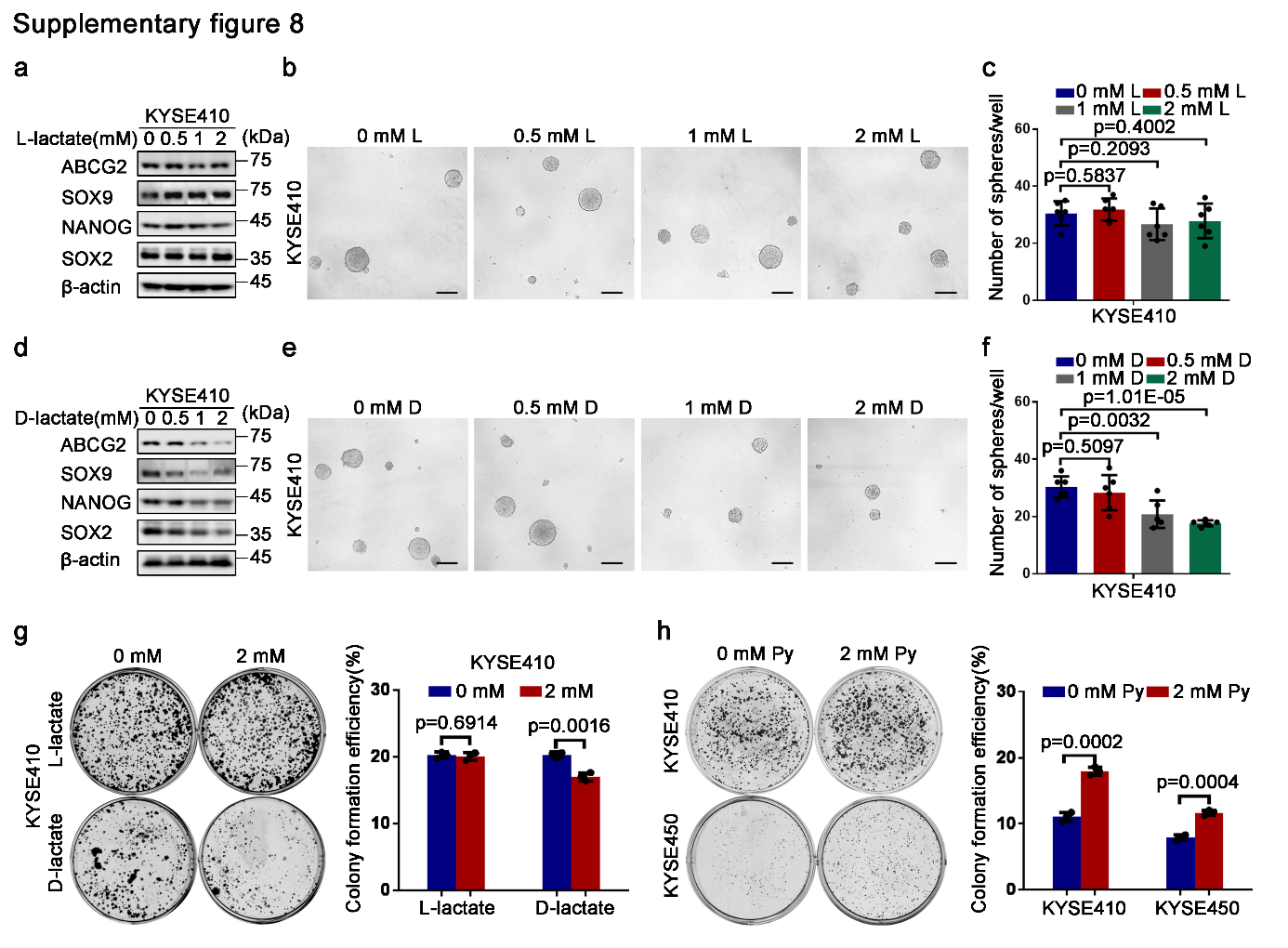


Figure. S8. D-lactate oxidation and pyruvate generation driven by CDK7-YAP-LDHD axis facilitate tumor cells stemness-related functions in ESCC.

**a** Western blot analysis showing the expression of stemness-related molecules in KYSE410 cells treated with indicated concentration of L-lactate for 72 h. **b** Representative micrographs showing the spheres formed by KYSE410 cells under the action of indicated concentration of L-lactate (L). **c** Histogram exhibiting the spheroids forming efficiency of KYSE410 cells treated with L-lactate (L). **d** Immunoblotting analysis displaying the expression of stemness-related molecules in KYSE410 cells treated with D-lactate at indicated concentration for 72 h. **e** Representative micrographs suggesting spheres formed by KYSE410 cells treated with D-lactate (D) at indicated concentration and (**f**) the spheres with a diameter of more than 100 μm were counted and graphed as a histogram. **g** Representative images suggesting the impact of 2 mM L-lactate and 2 mM D-lactate on colonies formed by KYSE410 cells (left) and these colonies were counted and graphed as a histogram (right). **h** Photographs illustrating the colonies formed by KYSE410 and KYSE450 cells treatment with pyruvate (Py) at indicated concentration (left) and the corresponding colony formation efficiency was presented as the histogram (right). Scale bars represent 100 μm in (**b**) and (**e**). Error bars represent mean ± SD (n=3).


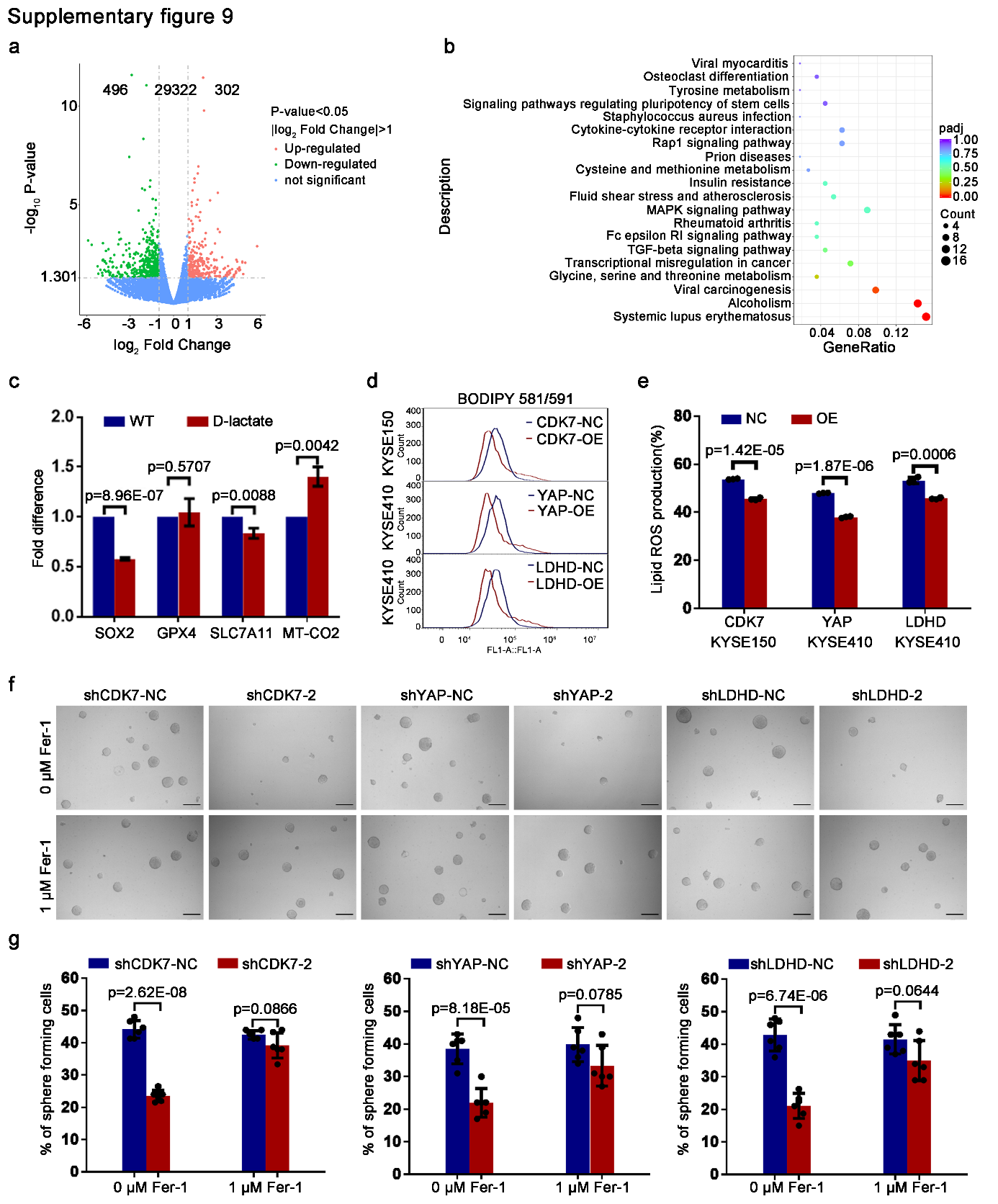


Figure. S9. D-lactate elimination mediated by CDK7-YAP-LDHD axis suppresses ferroptosis of ESCC cells.

**a** Volcano plot showing the differentially expressed genes in KYSE410 cells treated with 8 mM D-lactate for 72 h. **b** Dot plot indicating down-regulated signaling pathways after adding 8 mM D-lactate to KYSE410 cells. **c** RNA-seq analysis showing the changed mRNA levels of SOX2 and ferroptosis-related genes in KYSE410 cells treated with 8 mM D-lactate (n=3). **d** Flow cytometric analysis showing lipid ROS status through staining ESCC cells with 2 μM BODIPY 581/591 for 30 min. **e** Histogram illustrating relative lipid ROS generation when CDK7-YAP-LDHD axis was up-regulated in ESCC cells (n=3). **f** Representative photographs showing the spheres formed by CDK7, YAP or LDHD-depleted and their control KYSE410 cells treated with 1 μM Fer-1. Scale bar, 100 μm. **g** Histograms suggesting the spheres-forming efficiencies when CDK7-YAP-LDHD axis was down-regulated in the presence of Fer-1 (n=6). Error bars represent mean ± SD.

Table S1. shRNA sequences used in this study.

| shRNA | shRNA sequence (5’ to 3’) |
| --- | --- |
| shCDK7-1 | GCTGTAGAAGTGAGTTTGTAACTCGAGTTACAAACTCACTTCTACAGCTTTTT |
| shCDK7-2 | GAAACTGATCTAGAGGTTATACTCGAGTATAACCTCTAGATCAGTTTCTTTTT |
| shCDK7-NC | TTCTCCGAACGTGTCACGTTTCAAGAGAACGTGACACGTTCGGAGAATTTTTT |
| shYAP-1 | TTCTTTATCTAGCTTGGTGGC |
| shYAP-2 | TTTGGTTGATAGTATCACCTG |
| shYAP-NC | TTCTCCGAACGTGTCACGTTTCAAGAGAACGTGACACGTTCGGAGAATTTTTT |
| shLDHD-1 | CCTCATGAATCCAGGCAAAGT |
| shLDHD-2 | CAGACCAAGGAGGATCTGAAT |
| shLDHD-3 | CAACAGGTACAGCAAGCTGAA |
| shLDHD-NC | TTCTCCGAACGTGTCACGT |

Table S2. siRNA sequences used in this study.

| siRNA | target sequence (5’ to 3’) |
| --- | --- |
| LATS1 | GAACCAAACTCTCAAACAA |
| LATS2 | GTTCGGACCTTATCAGAAA |
| TEAD1/3/4 | GATCAACTTCATCCACAAGCT |
| TEAD2 | GCGAGTACCTGGTGAATTT |
| LDHD-1 | GCGTTAACCTGACGCATAT |
| LDHD-2 | GAAGCATTGTCGGGCATGT |
| LDHD-3 | GTGTGCCCATCATCCCATT |

Table S3. Primer sequences used in this study.

| Primer | Forward sequence (5’ to 3’) | Reverse sequence (5’ to 3’) |
| --- | --- | --- |
| CDK7 | ATGGCTCTGGACGTGAAGTCT | GCGACAATTTGGTTGGTGTTC |
| YAP | CCTCGTTTTGCCATGAACCAG | GTTCTTGCTGTTTCAGCCGCAG |
| NANOG | AATACCTCAGCCTCCAGCAGATG | TGCGTCACACCATTGCTATTCTTC |
| OCT4 | GACAACAATGAAAATCTTCAGGAGA | CTGGCGCCGGTTACAGAACCA |
| SOX2 | TGGACAGTTACGCGCACAT | CGAGTAGGACATGCTGTAGGT |
| SOX9 | AGCGAACGCACATCAAGAC | CTGTAGGCGATCTGTTGGGG |
| CTGF | AAAAGTGCATCCGTACTCCCA | CCGTCGGTACATACTCCACAG |
| CYR61 | AGCCTCGCATCCTATACAACC | TTCTTTCACAAGGCGGCACTC |
| LDHA | TTGGTCCAGCGTAACGTGAAC | CCAGGATGTGTAGCCTTTGAG |
| LDHB | GATGGATTTTGGGGGAACAT | AACACCTGCCACATTCACAC |
| LDHC | CCCTTGTTGATGTTGCATTGGA | ACGGAAACGGGCAGAGTCTA |
| LDHD | CAGGGTCAAGGCTTTTGCAG | TCACAGCACTTTGCCTGGAT |
| GAPDH | GGAGCGAGATCCCTCCAAAAT | GGCTGTTGTCATACTTCTCATGG |

Table S4. Antibodies Used in Western Blots (WB) and immunofluorescence (IF).

| Name | Vendor | Catalog No. | WB | IF |
| --- | --- | --- | --- | --- |
| CDK7 | Abcam | ab243863 | 1:1000 | 1:100 |
| YAP | CST | 14074S | 1:1000 | 1:100 |
| TEAD1 | Abcam | ab133533 | 1:1000 |  |
| TEAD2 | Proteintech | 21159-1-AP | 1:500 |  |
| TEAD4 | Abcam | ab197589 | 1:1000 |  |
| phospho-YAP (ser127) | CST | 4911 | 1:1000 | 1:50 |
| phospho-YAP (ser397) | CST | 13619 | 1:1000 | 1:50 |
| MST1 | CST | 3682 | 1:1000 |  |
| LATS1 | CST | 3477 | 1:1000 |  |
| LATS2 | CST | 5888 | 1:1000 |  |
| p-LATS1/2 | Immunoway | YP1222 | 1:1000 |  |
| phospho-RNAP II CTD(Ser2) | Bethyl | A300-654A | 1:1000 |  |
| phospho-RNAP II CTD(Ser5) | Millipore | 04-1572 | 1:1000 |  |
| SOX2 | CST | 3579S | 1:1000 |  |
| NANOG | CST | 4903S | 1:1000 |  |
| ABCG2 | Abcam | Ab108312 | 1:2000 |  |
| OCT4 | CST | 2750S | 1:1000 |  |
| SOX9 | CST | 82630T | 1:1000 |  |
| LDHA | Proteintech | 19987-1-AP | 1:2000 |  |
| LDHB | Proteintech | 19988-1-AP | 1:1000 |  |
| LDHC | Proteintech | 19989-1-AP | 1:1000 |  |
| LDHD | Proteintech | 14398-1-AP | 1:2000 | 1:100 |
| COX2 | CST | 12282 | 1:1000 |  |
| xCT | Proteintech | 26864-1-AP | 1:1000 |  |
| GPX4 | CST | 52455 | 1:1000 |  |
| GAPDH | Bioworld | AP0063 | 1:10000 |  |
| β-actin | Abcam | Ab8226 | 1:5000 |  |
| MitoTracker Red CMXRos | ThermoFisherScientific | M7512 |  | 1:5000 |
